# IGM: Integrated Gene-expression Modeling for multi-condition flux-preserving genome-scale metabolic models

**DOI:** 10.1371/journal.pone.0342294

**Published:** 2026-02-09

**Authors:** Thummarat Paklao, Apichat Suratanee, Kitiporn Plaimas

**Affiliations:** 1 Advanced Virtual and Intelligent Computing (AVIC) Center, Department of Mathematics and Computer Science, Faculty of Science, Chulalongkorn University, Bangkok, Thailand; 2 Department of Mathematics, Faculty of Applied Science, King Mongkut’s University of Technology North Bangkok, Bangkok, Thailand; 3 Intelligent and Nonlinear Dynamic Innovations Research Center, Science and Technology Research Institute, King Mongkut’s University of Technology North Bangkok, Bangkok, Thailand; 4 Centre of Excellence in Mathematics, Ministry of Higher Education, Science, Research, and Innovation, National University of Sciences, Bangkok, Thailand; 5 Omics Sciences and Bioinformatics Center, Faculty of Science, Chulalongkorn University, Bangkok, Thailand; Federal University Dutse, NIGERIA

## Abstract

Genome-scale metabolic models (GEMs) are powerful tools for studying cellular metabolism, but conventional approaches such as Flux Balance Analysis (FBA) often yield ambiguous results due to the lack of consistent integration of condition-specific data across multiple experimental contexts. Existing methods for incorporating gene expression data into GEMs are typically limited to single-condition analyses, rely on arbitrary thresholds, or compromise the interpretability of flux predictions. Here, we present IGM: Integrated Gene-expression Modeling, a novel mixed-integer linear programming (MILP) framework that integrates gene expression data across multiple conditions into GEMs without binarization, thereby preserving flux units and enhancing biological relevance. IGM employs flux variability analysis (FVA) to define feasible flux ranges, integrates relative gene expression through gene–protein–reaction (GPR) rules, and minimizes the difference between fluxes and corresponding gene expression mappings. Evaluation on *Escherichia coli* (*E. coli*) metabolic models demonstrates that IGM significantly improves correlation with experimentally measured fluxes, reduces flux solution ambiguity, and provides higher predictive consistency across multiple conditions. Among its variants, IGM with L1 norm regularization achieves the highest accuracy. We also evaluated gene expression integration by comparing relative gene expression values with model-derived gene expression variables. Visual inspection and genome-scale correlation analysis revealed strong concordance across all genes, confirming that IGM effectively preserves transcriptomic patterns while filtering out genes irrelevant to flux-carrying reactions, thereby enhancing biological interpretability. Furthermore, we applied IGM to flux change analysis, where subsystem-level fluxes revealed distinct metabolic states. This analysis highlights IGM’s ability to integrate transcriptomic data into metabolic modeling in a condition-specific yet consistent manner, enabling biologically grounded predictions of metabolic adaptation. By capturing dynamic metabolic changes and improving predictive accuracy, IGM provides a robust framework for consistent, comparative, and multi-condition metabolic studies.

## Introduction

The development of genome-scale metabolic models (GEMs) has facilitated the study of a diverse range of organisms [1], including bacteria [2,3], archaea [4,5], and eukaryotes including animals [6,7], plants [8,9] and human [10]. GEMs have emerged as essential computational frameworks for simulating and analyzing cellular metabolism at a system level [2]. By incorporating biochemical, genetic, and physiological data, these models provide valuable insights into the complex interactions among genes, metabolic reactions, and metabolites within a given organism. GEMs enable the investigation of cellular behavior, prediction of metabolic capabilities, and exploration of gene functions under various environmental and genetic perturbations [11,12]. Their applications span multiple disciplines [1], including metabolic engineering [13–15], where they serve as key tools for optimizing microbial strains for industrial bioproduction. In agriculture, GEMs support the development of crops that can survive in different environments and achieve higher yields [16,17]. Additionally, GEMs contribute to drug discovery by identifying potential therapeutic targets and elucidating metabolic vulnerabilities in pathogenic organisms and cancer cells [18–21]. Despite these successes, most applications rely on generic or static models that do not fully incorporate condition-specific transcriptomic data or enable consistent integration across multiple conditions. This limits their ability to capture dynamic and comparative metabolic responses across diverse biological contexts.

A variety of computational techniques have been developed to analyze and refine GEMs to extract biologically meaningful insights. One of the most widely used approaches is flux balance analysis (FBA) [22], a constraint-based optimization method that predicts the steady-state flux distribution of metabolic reactions based on mass balance. By simulating different environmental or genetic conditions, FBA can predict growth rates, identify essential genes, and optimize metabolic pathways for industrial applications [22–24]. Beyond standard FBA, several extensions have been introduced to improve metabolic predictions, including flux variability analysis (FVA) [25], which determines the range of feasible flux values, and parsimonious FBA (pFBA), which minimizes total flux while maintaining optimal growth or product formation [26]. However, a major limitation of these approaches is their inherent dependence on the initial reaction flux constraints and the choice of an objective function, both of which can introduce significant variability in the predicted flux values [27,28]. Because multiple flux distributions can achieve the same optimal objective value, traditional constraint-based models often fail to capture the actual physiological distribution of metabolic fluxes under specific biological conditions. This limitation becomes particularly evident when studying heterogeneous or dynamic metabolic states, where the underlying flux distributions may deviate from the singular optimal solution produced by conventional optimization techniques [28]. To overcome these limitations, several approaches have sought to integrate gene expression data into GEMs, thereby tailoring flux predictions to specific biological conditions. However, existing methods typically focus on single-condition analyses, often rely on arbitrary thresholding of expression values, or require binarization of gene activity. These strategies compromise both the interpretability and the consistency of resulting flux predictions. Importantly, they cannot simultaneously account for multiple experimental conditions within a unified framework, which limits their capacity to reveal comparative insights and dynamic metabolic adaptations.

Advances in high-throughput technologies such as microarrays and RNA sequencing (RNA-seq) have enabled systematic quantification of gene expression across multiple conditions [29]. Integrating this transcriptomic information into GEMs is crucial for generating condition-specific and biologically relevant flux predictions.

FBA has been enhanced by integrating gene expression data into either the objective function or the constraints, which each approach offering distinct advantages and limitations [30]. Two main categories of algorithms have been developed for incorporating gene expression into metabolic models. The first category includes methods that rely on single-condition data to estimate reaction fluxes. Examples include Gene Inactivity Moderated by Metabolism and Expression (GIMME) [31] and the Integrative Metabolic Analysis Tool (iMAT) [32], which use user-defined thresholds to binarize gene expression data. The E-flux method [33], in contrast, directly applies gene expression values to define reaction flux bounds. Its improved version, E-flux2 [34], extends this by incorporating the E-flux objective value as a constraint while redefining the objective function as the L2 norm of reaction flux variables. However, both E-flux and E-flux2 require prior knowledge to define an appropriate objective function. Another method, Simplified Pearson correlation with Transcriptomic data (SPOT) [34], avoids this requirement by maximizing the correlation between gene expression values and reaction fluxes. Nonetheless, SPOT generally performs worse than other approaches. Although E-flux, E-flux2, and SPOT directly integrate gene expression data, they do not preserve the units of reaction fluxes, complicating direct flux comparisons. To address this, Linear Bound Flux Balance Analysis (LBFBA) [35] was developed, which integrates gene expression data while maintaining flux units. However, LBFBA requires a large number of paired reaction fluxes and gene expression measurements to estimate the parameters for training linear flux bounds, which are then applied to constrain reaction fluxes. All methods in this category rely exclusively on single-condition gene expression data, limiting their utility for comparative or dynamic studies.

The second category of methods integrates gene expression data across multi-conditions. Metabolic Adjustment by Differential Expression (MADE) [36] incorporates differential gene expression into the model by converting expression values into binary form based on *p*-values. However, it struggles to accurately represent binary expression patterns, particularly when gene expression increases over successive intervals. Another method, Relative Expression and Metabolomic Integration (REMI) [37], calculates reaction fluxes for two conditions by integrating relative gene expression and metabolic data, enabling direct comparison of relative fluxes between these conditions. More recently, ICON-GEMs [38] was proposed, which incorporates gene co-expression network relationships into GEMs. While this approach improves performance under dynamic conditions compared to expression-only methods, it involves solving a non-convex quadratic programming problem, which is computationally demanding and may yield only local solutions. Taken together, current methods either (i) rely on single-condition data with arbitrary thresholds, (ii) compromise flux interpretability by not preserving flux units, or (iii) lack the ability to provide consistent predictions across multiple conditions in a unified framework.

To address these limitations, we propose IGM: Integrated Gene-expression Modeling, a novel mixed-integer linear programming (MILP) framework for the consistent integration of gene expression data across multiple conditions into genome-scale metabolic models. Unlike threshold-based single-condition methods, IGM directly incorporates relative gene expression without binarization, preserves flux units, and jointly constrains multiple conditions within a single optimization framework. We benchmarked IGM against conventional FBA, which does not incorporate gene expression, and against two representative single-condition integration methods—GIMME and E-flux. These methods represent gene-level information using either binary (GIMME) or continuous (E-flux) values. Although existing multi-condition methods have been proposed, their applicability is limited by data requirements and modeling assumptions, IGM inherently overcomes key limitations of these approaches. In particular, it avoids the restrictive binary representation of gene expression used in MADE and eliminates the inconsistent flux predictions that can arise in REMI when each condition must be solved separately. By integrating relative gene expression across conditions in a single coherent model, IGM provides more biologically interpretable, accurate, and comparable predictions of condition-specific flux distributions.

### IGM method overview

**Fig 1:**
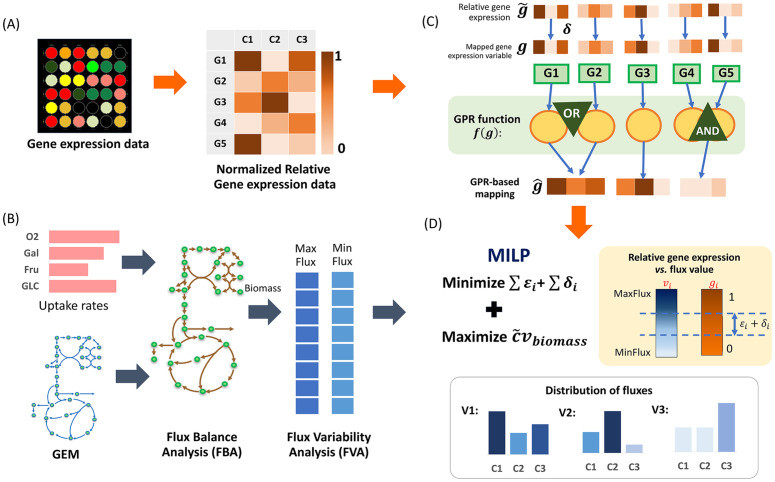
Workflow of IGM framework. **(A)** Gene expression data are transformed into a table of normalized relative expression values, ranging from 0 to 1. **(B)** Uptake rates and the GEM are provided as inputs for flux balance analysis (FBA) and flux variability analysis (FVA). **(C)** Relative gene expression values are mapped to expression variables and processed through gene–protein–reaction (GPR) rules to associate gene expression with reactions. **(D)** The MILP formulation of IGM determines the flux distribution for each condition by minimizing the difference between relative fluxes and relative gene expression while maximizing biomass production.

IGM integrates three primary inputs, which are a genome-scale metabolic model (GEM), measured carbon source uptake rates, and preprocessed, normalized relative gene expression data, as shown in Fig 1A,1B). Relative gene expression is mapped to reactions through gene-protein-reaction (GPR) rules based on the GPR function (*f*) (Fig 1C; see the Material and Methods section for details). Flux balance analysis (FBA) [22] is first applied to estimate maximum biomass production, followed by flux variability analysis (FVA) [25] to define feasible ranges for reaction fluxes. Finally, gene expression constraints are incorporated in a MILP formulation (Fig 1D), which minimizes the discrepancies between gene expression and corresponding reaction fluxes, while maximizing biomass. This integration refines flux predictions to better capture transcriptional regulation and condition-specific metabolic states. Our IGM avoids binarization, preserves flux units, and jointly analyzes multiple conditions within a single optimization framework. We also extend IGM with L1 and L2 norm regularization to improve predictive performance. Using *Escherichia coli* datasets, we demonstrate that IGM improves correlation with experimentally measured fluxes, reduces ambiguity in flux solutions, and provides more consistent and biologically interpretable predictions compared to both standard FBA and single-condition integration methods.

A full description of the MILP formulation and implementation of IGM is provided in the Material and Methods section.

## Materials and methods

### Datasets

We evaluated our approach using *Escherichia coli* (*E. coli*), a widely studied model bacterium. The latest GEM of *E. coli* is called iML1515 [3], includes metabolites, reactions, and genes. Our method uses transcriptomic data, fluxomic data, and initial fluxes as inputs, while experimentally measured fluxes obtained from ^13^C metabolic flux analysis (MFA) are used for validation. Three datasets were selected to demonstrate the performance of our algorithm:

(i) Data-A: Genetic and environmental perturbations (Ishii et al. [39])

This dataset investigates the metabolic response of *E. coli* to genetic (single-gene deletions) and environmental perturbations (dilution rates). It provides ^13^C -based flux data and RT-PCR mRNA abundances for the central carbon metabolism, pentose phosphate pathway (PPP), and the tricarboxylic acid (TCA) cycle in wild-type K12 *E. coli* growth in chemostat culture under different dilution rates (0.2, 0.5, and 0.7 h^-1^) and in five single-gene deletion strains (*pgm*, *pgi*, *gapC*, *zwf* and *rpe*).

(ii) Data-B: Growth on multiple carbon sources (Gerosa et al. [40])

The second dataset examines *E. coli* growth on eight different carbon sources: acetate, fructose, galactose, glucose, glycerol, gluconate, pyruvate and succinate [40]. Transcriptomic data was obtained from ArrayExpress (E-MTAB-3392).

(iii) Data-C: Strain-specific transcriptomic profiling (Holm et al. [41])

The third dataset involves transcriptome profiling of *E. coli* strains grown in MOPS medium supplemented with glucose during the mid-exponential phase. It includes three strains: a reference strain, an NADH oxidase mutant, and an ATPase mutant [41].

Details of the datasets used in this study, including transcriptomic data, uptake rates, measured fluxes, and the genome-scale metabolic model of *E. coli* are provided in S2 File (S1 Table).

### IGM formulation and implementation

The proposed method, IGM (Integrated Gene-expression Modeling), is a constraint-based modeling approach formulated as a mixed integer linear programming (MILP) problem built upon flux balance analysis (FBA) [22]. Flux balance analysis (FBA) is a linear programming technique widely used for analyzing metabolic systems. It estimates steady-state flux distributions across a metabolic network by optimizing a user-defined objective function (e.g., biomass production) subject to stoichiometric and flux constraints derived from experimental data.

The IGM objective combines two components which are maximizing biomass flux (as in standard FBA [22]), where $\tilde{c}$ denotes the biomass reaction coefficient, and minimizing the deviation between flux proportions and the corresponding relative gene expression levels based on gene-protein-reaction (GPR) associations. This deviation is quantified using non-negative variables $\delta_{i}^{\text{1}},\;\;\delta_{i}^{\text{2}},\;\;\varepsilon_{j}^{\text{1}},\;$ and $\varepsilon_{j}^{\text{2}}$ for i=1,2,3,…, k (genes) and j=1,2,3,…,n (reactions). These variables measure the distance between relative gene expression and flux values in the optimization. Thus, the IGM objective function in Eq (1) minimizes a weighted combination of these deviation terms while maximizing biomass production, subject to steady-state, flux bound, and mixed integer constraints. The complete IGM formulation, including its objective function and constraints, is presented as follows in Eqs (1)–(13).


$$Minimize\;-\tilde{c}v_{biomass}+\sum_{i=\text{1}}^{k}\left(\delta_{i}^{\text{1}}+\delta_{i}^{\text{2}}\right)+\sum_{j=\text{1}}^{n}\left(\varepsilon_{j}^{\text{1}}+\varepsilon_{j}^{\text{2}}\right)$$ (1)

$$\text{Subject}\;\text{to}\sum_{j=\text{1}}^{n}s_{ij}v_{j}=\text{0}\;\text{for}\;i=\text{1},\;\text{2},\;\text{3},\ldots ,\;m$$ (2)

$$L_{j}\leq v_{j}\leq U_{j}*b_{j}\;\text{for}\;\text{all}\;j=\text{1},\;\text{2},\;\text{3},\;\ldots ,\;n$$ (3)

$$v_{j}\leq v_{j}^{exp}\;\text{for}\;\text{all}\;j\in EXP$$ (4)

$$b_{i}+b_{j}=\text{1}\;\text{for}\;\text{all}\;(i,j)\in RE$$ (5)

$$\frac{v_{j}-\underset{d}{\text{min}}\left\{\alpha_{j,d}\right\}}{\underset{d}{\text{max}}\left\{\beta_{j,d}\right\}-\underset{d}{\text{min}}\left\{\alpha_{j,d}\right\}}={\hat{g}}_{j}+\varepsilon_{j}^{\text{1}}-\varepsilon_{j}^{\text{2}}\;\text{for}\;\text{all}\;j=\text{1},\;\text{2},\;\text{3},\;\ldots ,\;n$$ (6)

$${\hat{g}}_{j}=f(\left\{g_{i}\right|\;i\in G_{j}\})\;\text{for}\;\text{all}\;j=\text{1},\;\text{2},\;\text{3},\;\ldots ,\;n,$$ (7)

$$g_{i}-{\tilde{g}}_{i}=\delta_{i}^{\text{1}}-\delta_{i}^{\text{2}}\;\text{for}\;\text{all}\;i=\text{1},\;\text{2},\;\text{3},\;\ldots ,\;k$$ (8)

$$\text{0}\leq {\hat{g}}_{j}\leq \text{1}\;\text{for}\;\text{all}\;j=\text{1},\;\text{2},\;\text{3},\;\ldots ,\;n$$ (9)

$$\text{0}\leq g_{i}\leq \text{1}\;\text{for}\;\text{all}\;i=\text{1},\;\text{2},\;\text{3},\;\ldots ,\;k$$ (10)

$$\varepsilon_{j}^{\text{1}},\varepsilon_{j}^{\text{2}}\geq \text{0}\;\text{for}\;\text{all}\;j=\text{1},\;\text{2},\;\text{3},\;\ldots ,\;n$$ (11)

$$\delta_{i}^{\text{1}},\;\delta_{i}^{\text{2}}\geq \;\text{0}\;\text{for}\;\text{all}\;i=\text{1},\;\text{2},\;\text{3},\;\ldots ,\;k$$ (12)

$$b_{j}\in \left\{\text{0},\;\text{1}\right\}\;\text{for}\;\text{all}\;j=\text{1},\;\text{2},\;\text{3},\;\ldots ,\;n$$ (13)

Here, $\alpha_{j,d}$ and $\beta_{j,d}$ represent the minimum and maximum possible fluxes of reaction j under condition d, as determined by FVA. The variable ${\hat{g}}_{j}$ denotes the relative gene expression variable for reaction j derived from the gene-protein-reaction (GPR) rules, where f(·) is the GPR function and $G_{j}$ is the set of genes associated with reaction *j*, formulated as a mixed-integer constraint representing the GPR associations. The variable $g_{i}$ corresponds to the expression level of gene i in the model, while both ${\hat{g}}_{j}$ and $g_{i}$ are normalized to the range [0, 1]. Finally, ${\tilde{g}}_{i}$ denotes the relative gene expression value of gene i (precomputed from raw data). A schematic illustration of $\tilde{g},\;g,$ and $\hat{g}$ is provided in Fig 1C. First, the constant relative gene expression values ${\tilde{g}}_{i}$ are computed by transforming the gene expression data using Eqs. (15)–(18). The variable $g_{i}$ is the model-state gene expression mapped from ${\tilde{g}}_{i}$ through Eq (8), which may vary slightly. Finally, ${\hat{g}}_{j}$ is enzyme expression/activity inferred from GPR rules using Eqs (19)–(25) via GPR function f(·), as shown in Eq (7). In our framework, the relative gene expression values ${\tilde{g}}_{i}$ are not used directly as fixed reaction-level scores. Instead, ${\tilde{g}}_{i}$ serves as a reference that is mapped to the internal model variable $g_{i}$ through Eq (8). The variable $g_{i}$ is allowed to vary within the optimization problem so that the model can flexibly adjust gene–reaction contributions to best fit the flux constraints and the data (Additional details can be found in S1 File).

The standard FBA constraints are shown in Eqs (2)–(5). Consider an irreversible GEM containing m metabolites and n reactions, where all reaction fluxes are constrained to be non-negative. Let [M] be a vector of metabolite concentrations of length m. The rate of change of metabolite concentration can be described in a system of differential equation ($\frac{d[M]}{dt}=S\cdot v$, where $S\;\in R^{m\times n}$ is a stoichiometric matrix, $v\in R^{n}$ is the vector of reaction fluxes). At steady state, the system satisfies $\;\frac{d[M]}{dt}=\text{0}.\;$ Therefore, Sv=0. This condition ensures that, for each metabolite, the total production rate equals the total consumption rate, maintaining mass balance throughout the network.

To identify the reaction flux vector v in the system Sv=0, one could directly solve the linear equation system. However, in most cases, the number of reactions exceeds the number of metabolites, resulting in underdetermined system with no unique solution. Therefore, optimization techniques are employed to identify and analyze optimal flux distribution within a constrained solution space. The constraints include steady-state mass balance, as shown in Eq (2), ensuring metabolite concentrations remain constant at steady state, flux bounds, as in Eq (3), where upper and lower limits are imposed on reaction fluxes based on experimental data. Here, $L_{j}$ and $U_{j}$ represent the lower and upper bounds, respectively, for reaction flux j. Since all reactions are irreversible, $L_{j}=\text{0}$ for all reaction j. A binary variable $b_{j}\in \{\text{0},\;\text{1}\}$ is introduced to represent the activity state of reaction j. Since we assume an irreversible model, all fluxes must be non-negative. For reversible reactions decomposed into two irreversible reactions, only one can be active at a time. This is enforced by binary constraints, as in Eqs (5) and (13). If $b_{j}=\text{0}$, the upper bound in Eq (3) is forced to zero, effectively shutting down the reaction. If $b_{j}=\text{1}$, the flux is constrained by its upper bound $U_{j}$. In practice, unknown upper bounds are set to a sufficiently large number (here, $U_{j}=\text{1},\text{0}\text{0}\text{0}$). Experimental flux data are incorporated by setting measured flux values as lower bounds for corresponding reactions, as defined in Eq (4), where EXP denotes the set of experimentally measured external reaction fluxes $v_{j}^{exp}$. RE represents the set of index pairs corresponding to irreversible reactions, obtained by splitting reversible reactions.

Constraint in Eq (6) establishes a direct relationship between the proportion of gene expression levels and the corresponding proportion of flux levels. Flux proportions are determined using a min-max scaling approach, where the minimum and maximum flux values are obtained from FVA across different conditions. This normalization ensures that the relative flux distributions are scaled consistently across varying metabolic states. Gene expression levels, which serve as regulatory inputs for flux constraints, are derived using the proposed computational approach for Gene-Protein-Reaction (GPR) representation. The computed gene expression levels and their GPR associations are incorporated into the optimization model through constraint in Eq (7). Furthermore, normalized relative gene expression values are integrated the optimization framework by minimizing the difference between these values and the gene variables, as formulated in Eq (8). These transformations ensure the effective alignment between gene expression constraints and flux constraints, thereby enabling biologically meaningful integration of transcriptomic into the metabolic model. Constraints in Eqs (9)–(13) enforce the non-negativity and binary conditions on specific variables, ensuring that metabolic fluxes and gene expression-related variables remain within biologically realistic and computationally feasible bounds. Moreover, the matrix formulation of the IGM optimization problem is provided in S3 Text in S1 File.

Additionally, we extend IGM through a two-step optimization process. In the first step, we compute IGM. In the second step, we formulate a model with L1 and L2 norms regularizations as the objective function, incorporating the optimal value from IGM as a constraint, as shown in Eq (14).


$$-\tilde{c}v_{biomass}+\sum_{i=\text{1}}^{k}\left(\delta_{i}^{\text{1}}+\delta_{i}^{\text{2}}\right)+\sum_{j=\text{1}}^{n}\left(\varepsilon_{j}^{\text{1}}+\varepsilon_{j}^{\text{2}}\right)={\hat{z}}^{*},$$ (14)

where ${\hat{z}}^{*}$ is the optimal objective value obtained from IGM. The extended version of IGM with the L1 norm, so-called IGM + L1, employs the L1 norm by minimizing $\sum_{j=\text{1}}^{n}v_{j}$, which promotes sparsity in the flux distribution by encouraging only a subset of reactions to carry flux, subject to the constraints from Eqs (2)–(13) and Eq (14). Conversely, IGM + L2 employs the L2 norm by minimizing $\sum_{j=\text{1}}^{n}v_{j}^{\text{2}}$, which penalizes excessively large flux values without driving them to zero, resulting in a smoother and more evenly distributed flux profile. Similar to IGM + L1, its constraints are given by Eqs (2)–(13) and Eq (14). The IGM algorithm is implemented in MATLAB and is publicly available at https://github.com/ThummaratPaklao/IGM, relying on the COBRA Toolbox [42,43].

### Flux variability analysis

Since the programming of several constraint-based models can yield alternative optimal solutions, flux variability analysis (FVA) [25] is proposed to identify the range of possible reaction fluxes. FVA is also a constraint-based method used to evaluate the minimum and maximum flux of each reaction in the metabolic model while still achieving a defined value of the objective function from FBA. The objective of FVA is to minimize and maximize each reaction flux $v_{i}$. The possible minimum and maximum fluxes of reaction $v_{i}$ under condition d are represented as $\alpha_{i,d}$ and $\beta_{i,d}$, respectively. The constraints used in FVA are the same as those in FBA, as described in the previous section, but FVA includes an additional constraint: the objective value obtained from FBA, $\sum_{j=\text{1}}^{n}c_{j}v_{j}={pf}^{*}$, is fixed as a constraint, where $f^{*}$ is the optimal solution of the original FBA optimization, p∈[0,1] represents the proportion of $f^{*}$, and $c_{j}$ denotes the contribution of the fluxes of interest.

### Gene expression transformation

To integrate the relative gene expression data into metabolic models, we need to transform the gene expression values relative to other conditions into a range between 0 and 1. Let ${\tilde{g}}_{i,j}$ represent the transformed relative gene expression value of gene i in condition j. We propose three transformation equations. The first approach involves dividing the gene expression value by the maximum of gene expression across conditions as a reference, as shown in Eq (15).


$${\tilde{g}}_{i,j}=\frac{T_{i,j}}{\underset{j}{\text{max}}\left\{T_{i,j}\right\}},$$ (15)

where $T_{i,j}$ is the gene expression value of gene i in condition j, derived from transcriptomic profiles.

The second approach uses a min-max scaling technique. This method calculates the ratio between the difference (distant) of the gene expression value and the minimum gene expression value across conditions, and the range between the maximum and minimum gene expression values across conditions. The min-max scaling formula is shown in Eq (16), as follows.


$${\tilde{g}}_{i,j}=\frac{T_{i,j}-\underset{j}{\text{min}}\left\{T_{i,j}\right\}}{\underset{j}{\text{max}}\left\{T_{i,j}\right\}-\underset{j}{\text{min}}\left\{T_{i,j}\right\}}.$$ (16)

The final proposed transformation accounts for the distribution of gene expression data across different conditions by using the mean gene expression value, ${\overset{―}{T}}_{i}$, as a reference, which is scaled to 0.5, as shown in Eqs (17) and (18). When $T_{i,j}={\overset{―}{T}}_{i}$, the transformed relative gene expression ${\tilde{g}}_{i,j}$ becomes 0.5, representing the midpoint between 0 and 1. For $T_{i,j}>{\overset{―}{T}}_{i}$, the transformation follows Eq (17), which applies min-max scaling by treating ${\overset{―}{T}}_{i}$ as the minimum. The result is divided by two, mapping the range to (0, 0.5], and then 0.5 is added to shift the range to (0.5, 1], ensuring values greater than 0.5. Conversely, when $T_{i,j}<{\overset{―}{T}}_{i}$, Eq (18) is used, where ${\overset{―}{T}}_{i}$ serves as the maximum in min-max scaling. The scaled value is then divided by two, resulting in values in the range [0, 0.5).


$${\tilde{g}}_{i,j}=\frac{T_{i,j}-\overset{―}{T_{i}}}{\text{2}(\underset{j}{\text{max}}\left\{T_{i,j}\right\}-\overset{―}{T_{i}})}+\frac{\text{1}}{\text{2}}\;\text{if}\;T_{i,j}\geq \overset{―}{T_{i}};$$ (17)

$${\tilde{g}}_{i,j}=\frac{{\overset{―}{T}}_{i}-T_{i,j}}{\text{2}({\overset{―}{T}}_{i}-\underset{j}{\text{min}}\left\{T_{i,j}\right\})},\;\text{otherwise}.$$ (18)

Here ${\overset{―}{T}}_{i}$ represents the average of gene expression value of gene i, and $\underset{j}{\text{max}}\left\{T_{i,j}\right\}-\underset{j}{\text{min}}\left\{T_{i,j}\right\}>\omega$ in Eq (16), $\underset{j}{\text{max}}\left\{T_{i,j}\right\}-\overset{―}{T_{i}}>\omega$ in Eq (17), and ${\overset{―}{T}}_{i}-\underset{j}{\text{min}}\left\{T_{i,j}\right\}>\omega$ in Eq (18). The parameter ω is small value introduced to avoid division by zero, and in this work, we set ω=0.001.

In our workflow, gene expression values are normalized prior to GPR evaluation. This normalization places each gene on a comparable scale [0, 1], preventing highly expressed genes from dominating the min/max aggregation in GPR rules and enabling the resulting reaction scores to reflect relative transcriptional activity rather than raw magnitude. Since the optimization model uses relative gene activity as a soft constraint, pre-normalization provides stable and interpretable inputs compared to approaches that compute GPR scores on raw expression and normalize afterward. Further discussion can be found in S1 Text in S1 File.

### Representation of gene-protein-reaction association using mixed-integer constraints

Gene-protein-reaction (GPR) associations describe the relationships among genes, proteins, and reactions using Boolean logic, expressed in terms of “AND” and “OR” rules. In an “AND” relationship within a GPR rule, an enzymatic complex is encoded by multiple genes, meaning all subunits are required for the reaction to carry flux. Conversely, when multiple genes encode isozymes, where different enzymes catalyze the same reaction, these are represented by an “OR” rule in the GPR expression. The GPR association is illustrated in S1 Fig within S1 File. This concept can be extended to continuous gene expression levels. For an “AND” relationship, the enzymatic activity is determined by the minimum expression level among the associated genes. For an “OR” relationship, either the summation or the maximum of the gene expression levels can be used. However, traditional approaches compute these gene expression values externally, outside of the optimization framework. To integrate these relationships directly into computational models, we introduce constraints that map gene expression values into a metabolic model. Let $g_{i}$ be the expression variable for gene i, constrained within the range [0, 1].


$${\hat{g}}_{j}=f\left(\left\{g_{i}\right|\;i\;\in G_{j}\}\right)=\left\{ \begin{array}{c} \underset{i\in R_{j}}{\text{min}}\left(g_{i}\right)\;\;\text{if}\;R_{j}\;\text{is}\;\text{an}\;\text{AND}\;\text{relationship}\\ \underset{i\in R_{j}}{\text{max}}\left(g_{i}\right)\text{if}\;R_{j}\;\text{is}\;\text{an}\;\text{OR}\;\text{relationship} \end{array},\right.\;$$ (19)

where ${\hat{g}}_{j}$ is the new variable introduced in the programming framework to represent the calculated gene expression level associated with reaction flux *j*. $G_{j}$ represents the set of genes associated with reaction *j*. The subset ${R_{j}\subseteq G}_{j}$ includes those genes participating in the specific GPR rule. For an AND relationship, the enzyme activity is constrained by the lowest expressed subunit; therefore, the minimum expression value is used. For an OR relationship, any gene suffices to express the enzyme, and the maximum expression value is used. The mapping function f links genes to reaction fluxes, and $R_{j}$, denotes the set of genes involved in the “AND” and “OR” relationships for reaction j.

Optimization solvers cannot directly handle constraints expressed using the min or max functions. To address this, we reformulate these functions using binary variables. For the min function of k genes, we introduce *k* binary variable $y_{i}\in \{\text{0},\;\text{1}\}$ to select the gene with the minimum expression value. The following 2k+1 constraints are introduced as follows:


$${\hat{g}}_{j}\leq g_{i}\;\text{for}\;\text{all}\;i\;\text{in}\;R_{j}\;\text{relationship};$$ (20)

$${\hat{g}}_{j}\geq g_{i}-My_{i}\;\text{for}\;\text{all}\;i\;\text{in}\;R_{j}\;\text{relationship};$$ (21)

$$\sum_{i\in R_{j}}y_{i}=k-\text{1},$$ (22)

where M is a sufficiently large constant. The first constraint ensures that ${\hat{g}}_{j}$ is not greater than any gene expression level. The second and third constraints ensure that exactly one $y_{i}$ is zero, thereby selecting the minimum gene expression value. When $y_{i}=\text{0}$, the second constrain enforces ${\hat{g}}_{j}\geq g_{i}$ and combined with the first constraint, this guarantees that ${\hat{g}}_{j}$ equals the minimum of $g_{i}$. Similarly, for the max function, we introduce k binary variables $y_{i}\in \{\text{0},\text{1}\}$ to select the gene with the maximum expression value, leading to the following constraints:


$${\hat{g}}_{j}\geq g_{i}\;\text{for}\;\text{all}\;i\;\text{in}\;R_{j}\;\text{relationship}$$ (23)

$${\hat{g}}_{j}\leq g_{i}+My_{i}\;\text{for}\;\text{all}\;i\;\text{in}\;R_{j}\;\text{relationship}$$ (24)

$$\sum_{i\in R_{j}}y_{i}=k-\text{1}$$ (25)

Here, the first constraint ensures ${\hat{g}}_{j}$ is greater than or equal to all related gene expression levels. The second and third constraints enforce selection of the maximum expression level. When $y_{i}=\text{0}$, the second constrain becomes ${\hat{g}}_{j}\leq g_{i}$ and combined with the first constraint, this ensures ${\hat{g}}_{j}$ equals the maximum of $g_{i}$. Details of the formulation for the gene–protein–reaction association function under complex rules are provided in S2 Text in S1 File.

### Accuracy measurement

(a) Uncentered Pearson product-moment correlation

The uncentered Pearson product-moment correlation is used to measure the linear relationship between two vectors, as follows:


$$R=\frac{\sum_{i=\text{1}}^{nme}v_{i}^{p}v_{i}^{m}}{\sqrt{\sum_{i=\text{1}}^{nme}{\left(v_{i}^{p}\right)}^{\text{2}}\sum_{i=\text{1}}^{nme}{\left(v_{i}^{m}\right)}^{\text{2}}}},$$ (26)

where $v_{i}^{p}$ and $\;v_{i}^{m}$ represent the predicted and measured fluxes for reaction *i*, respectively, and R is the uncentered Pearson correlation coefficient.

We apply this correlation coefficient between predicted fluxes and measured fluxes obtained from ^13^C metabolic flux analysis to assess the consistency of fluxes generated by our algorithm. This coefficient is convenient for comparison because its value lies in the range [−1, + 1]. A correlation coefficient close to +1 or −1 indicates a strong positive or negative linear correlation between predicted and measured fluxes, respectively. In contrast, a correlation coefficient near zero indicates no linear relationship between the predicted and measured flux.

However, measured fluxes cannot always be mapped one-to-one with predicted fluxes due to the complex interplay between metabolites and reactions in a metabolic network. These relationships can be represented using “AND” and “OR” logic. In an “AND” relationship, when a measured flux corresponds to multiple intermediate reactions connected sequentially, the minimum flux among these reactions is computed to represent the slowest step in the pathway. Conversely, in an “OR” relationship, when a measured flux corresponds to multiple parallel reactions, the summation of predicted fluxes is calculated, reflecting the higher overall flux values typically resulting from multiple alternative pathways.

(b) Normalized root mean square error

We also compute the normalized root mean square error (NRMSE) between predicted and measured fluxes to evaluate prediction accuracy. The NRMSE is calculated as


$$NRMSE=\;\frac{\Delta v_{i}-{\Delta v}_{i}^{min}}{{\Delta v}_{i}^{max}-{\Delta v}_{i}^{min}},$$ (27)

where $\Delta v_{i}$ is the root mean square error (RMSE) between predicted and measured fluxes, defined as


$$\Delta v_{i}=\sqrt{\sum_{i=\text{1}}^{t}{\left(v_{i}^{p}-v_{i}^{m}\right)}^{\text{2}}.}$$ (28)

Here, t is the number of measured fluxes, while ${\Delta v}_{i}^{max}$ and ${\Delta v}_{i}^{min}$ represent the maximum and minimum RMSE values, respectively.

### Flexibility of reaction fluxes

The flexibility of reaction fluxes is assessed by determining the minimum and maximum range of each flux in the metabolic model while ensuring that the objective function from the first-stage optimization remains well-defined. Since various constraint-based methods may yield optimal solutions, the flexibility of reaction i (${FR}_{i}$) is calculated using Eq (29) as


$${FR}_{i}=\left|v_{i}^{max}-v_{i}^{min}\right|,$$ (29)

where $v_{max}^{i}$ and $v_{min}^{i}$ represent the maximum and minimum fluxes for reaction i, respectively. These values are determined using Flux Variability Analysis (FVA), which employs a two-stage optimization approach to evaluate reaction flux flexibility. In the first stage, the relevant objective function of IGM, denoted as ${\hat{z}}^{*}$, is computed. In the second stage, a constraint-based modeling technique is applied, where the IGM objective function is incorporated as a constraint, and each reaction flux in the metabolic model is independently maximized and minimized.

## Results

We evaluated IGM using the *Escherichia coli* genome-scale metabolic model iML1515 [3], together with three associated gene expression datasets (described in the Dataset section). *E. coli* was chosen as a benchmark organism because it is well-studied and has experimentally measured fluxes from ^13^C metabolic flux analysis (MFA), which we use for validation. Three datasets cover different biological contexts: Data-A contains flux and transcriptomic measurements under genetic deletions and different dilution rates [39]. Data-B profiles gene expression during growth on eight distinct carbon sources [40], while Data-C provides strain-specific transcriptomes from a reference and two mutant strains [41]. More details on each dataset are provided in the Dataset section. The Results section (here) is organized into three main parts: (i) comparison with standard FBA, (ii) comparison with methods for integrating single-gene expression data, and (iii) analysis of function selection and parameter tuning.

### Comparative evaluation of IGM, FBA, and their regularized variants

**Fig 2:**
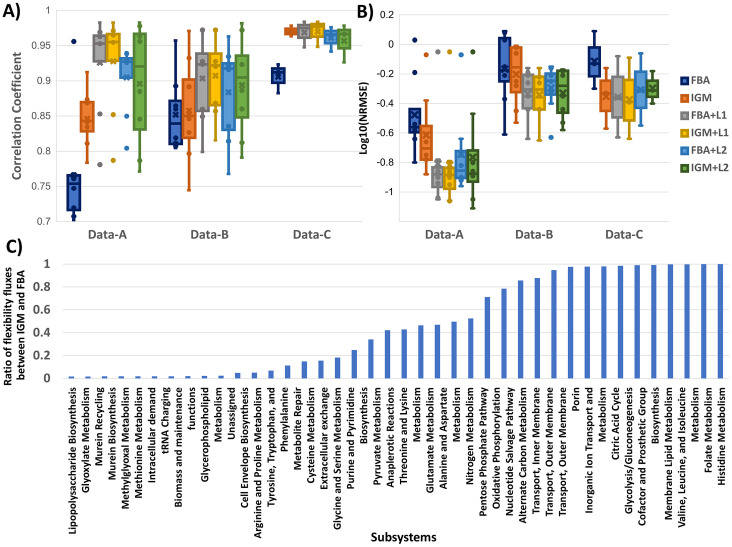
Performance comparison of IGM, FBA, and regularized variants. **(A-B)** Predictive accuracy of IGM, FBA, and their L1/L2 regularized variants, evaluated by correlation coefficient **(A)** and normalized root mean square error (NRMSE) **(B)** between predicted fluxes and experimentally measured fluxes across three *E. coli* datasets (Data-A, Data-B, and Data-C). IGM consistently shows higher correlation and lower NRMSE compared to FBA. Regularization further improves performance, with L1 yielding the most stable and accurate predictions. **(C)** Reaction flux flexibility ratio between IGM and FBA across metabolic subsystems. Ratios ≤ 1 indicate that IGM reduces or maintains solution space relative to FBA, thereby refining flux predictions and improving reliability.

We compare IGM, a method for integrating relative gene expression data into metabolic models, with FBA, the original flux balance analysis method that does not incorporate gene expression data. Additionally, we evaluate two extended versions of IGM, namely IGM + L1 (IGM with L1 norm) and IGM + L2 (IGM with L2 norm), which introduce different regularization techniques to improve predictive performance. For a fair comparison, we also include FBA + L1 norm (parsimonious FBA, or pFBA) and FBA + L2 norm as baseline extensions of FBA. To assess performance, we analyze the correlation coefficient and normalized root mean square error (NRMSE) between measured fluxes (obtained from ^13^C metabolic flux analysis) and predicted fluxes, as shown in Fig 2, where Fig 2A and Fig 2B demonstrate the distribution of correlation coefficients and NRMSE values across different datasets.

From Fig 2A and Fig 2B, we observe that IGM consistently outperforms FBA in predictive accuracy across all datasets. Specifically, IGM achieves higher average correlation coefficients and lower average NRMSE values compared to FBA, demonstrating that incorporating gene expression data significantly improves flux predictions. The correlation coefficients between predicted and measured fluxes for IGM exceed 0.75, 0.7, and 0.95 for Data-A, Data-B, and Data-C, respectively, indicating strong predictive performance under different conditions. Furthermore, adding L1 and L2 norms to both FBA and IGM further improves prediction accuracy. These regularization techniques increase correlation coefficients and decrease NRMSE values across all datasets. This result suggests that regularization, particularly L1 and L2 norms, enhances flux distribution predictions by reducing solution variability and enforcing sparsity (L1) or smoothness (L2) in flux profiles.

When considering overall performance, L1 regularization yields the best results, producing the highest average correlation coefficients and the lowest NRMSE values, with minimal standard deviation across datasets. This indicates that L1 regularization not only improves accuracy but also stabilizes predictions. In contrast, while L2 regularization also improves performance, it introduces higher variability, as reflected by a larger standard deviation across conditions.

Beyond predictive accuracy, we also examine reaction flux flexibility in IGM compared to FBA (Fig 2C). Because constraint-based models often yield multiple optimal solutions, reaction flux flexibility provides insight into the size of the feasible solution space. Higher flexibility implies a larger solution space, which can lead to greater variability in flux predictions and reduced reliability. Fig 2C shows a bar plot of the average reaction flux flexibility ratio between IGM and FBA across metabolic subsystems. A ratio ≤ 1 indicates that IGM has a smaller or equivalent solution space compared to FBA, whereas a ratio > 1 would indicate greater flexibility in IGM. From Fig 2C, most subsystems display a flexibility ratio < 1, demonstrating that IGM generally reduces the solution space relative to FBA. This reduction implies that incorporating gene expression constraints helps refine flux predictions and improve their reliability. A few subsystems exhibit a ratio equal to 1, indicating equivalent flexibility between IGM and FBA. Importantly, no subsystems exhibit a ratio greater than 1, confirming that IGM does not introduce excessive variability into the metabolic model.

### Comparative evaluation of IGM, extended IGM, and single-condition integration methods

**Fig 3:**
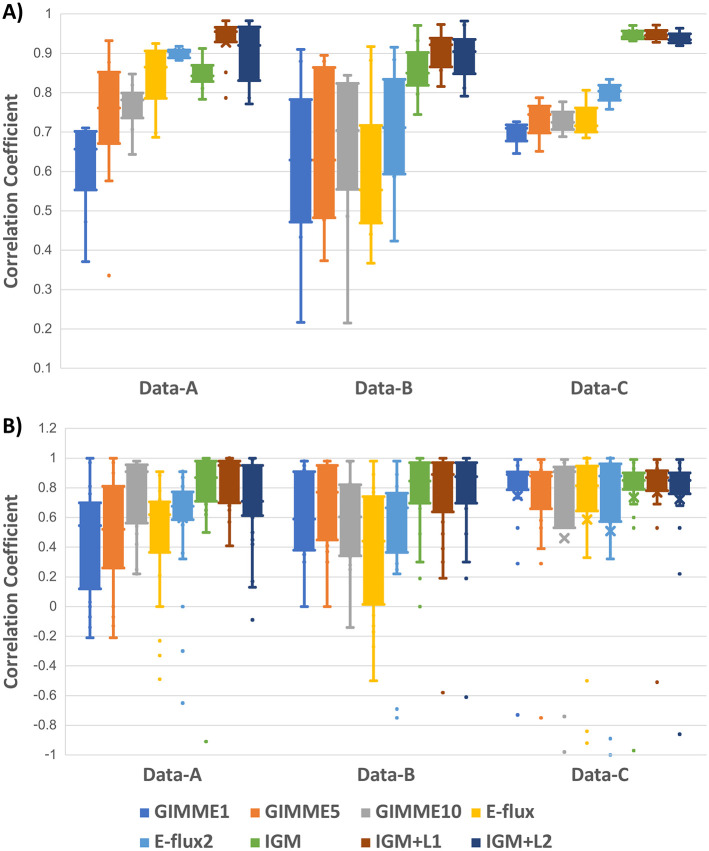
Comparison of IGM, extended IGM, and single-condition integration methods. **(A)** Correlation coefficients between predicted and measured fluxes within each condition for GIMME (various thresholds), E-flux, E-flux2, IGM, IGM + L1, and IGM + L2 across three *E. coli* datasets. Single-condition methods show variable performance, with E-flux2 outperforming GIMME and E-flux but remaining less consistent than IGM-based methods. **(B)** Correlation between measured fluxes and predicted fluxes across conditions. IGM and its regularized variants achieve higher cross-condition consistency than all single-condition methods, demonstrating their strength in capturing dynamic flux changes. Overall, IGM + L1 provides the best balance of accuracy and stability across datasets.

In this study, we compare IGM with several widely used single-condition integration methods. These include the GIMME algorithm (tested with varying thresholds) and the E-flux and E-flux2 approaches. We selected GIMME as a representative threshold-based approach, and E-flux and E-flux2 to represent continuous-value integration. These methods are well-established, extensively benchmarked, and provide a clear baseline for assessing improvements offered by multi-condition integration. Additionally, we evaluate the performance of IGM, IGM + L1, and IGM + L2 to assess the impact of incorporating different regularization techniques. To systematically compare these approaches, we analyze the correlation coefficient between predicted fluxes and measured fluxes, as illustrated in Fig 3A and 3B. Fig 3A presents the correlation between predicted and measured flux vectors within each condition, while Fig 3B focuses on the correlation between measured fluxes and their predicted values across multiple conditions. The key distinction between these two metrics is their interpretation: the first evaluates flux prediction consistency within a single condition, whereas the second examines flux variation across conditions.

Fig 3A shows boxplots of correlation coefficients for various methods, including single-condition approaches (GIMME, E-flux, and E-flux2), IGM, and extended IGM. The y-axis represents correlation coefficients, while the *x*-axis lists the algorithms applied to each dataset (Data-A, Data-B, and Data-C). The results indicate that GIMME exhibits highly variable performance depending on the chosen threshold. Since the threshold parameter determines how gene expression values are binarized (active/inactive), its selection directly affects reaction activity. For Data-A, increasing the threshold improves correlation, whereas in Data-B and Data-C, higher thresholds decrease performance, illustrating GIMME’s sensitivity to threshold choice and its potential limitations in practical applications. In contrast, E-flux and E-flux2, which do not rely on thresholds, consistently outperform GIMME across all datasets. Among these, E-flux2, an enhanced version of E-flux, yields a significantly higher correlation coefficient, demonstrating superior robustness and predictive accuracy.

We next compare single-condition methods to IGM, IGM + L1, and IGM + L2. IGM-based methods achieve significantly higher correlation coefficients than single-condition methods for Data-B and Data-C. Although E-flux2 slightly outperforms IGM in Data-A, IGM still exceeds the performance of GIMME and E-flux, highlighting its effectiveness in integrating multi-condition gene expression data. A closer examination reveals that both IGM + L1 and IGM + L2 outperform E-flux2, suggesting that incorporating norm-based regularization into IGM’s objective function improves predictive accuracy. Between the two, IGM + L1 delivers the best overall performance across datasets, underscoring the advantage of L1 regularization in stabilizing flux predictions.

Although IGM shows slightly lower within-condition correlation (Fig 3A) compared to E-flux2 for Data-A, it excels in capturing flux dynamics across conditions. Fig 3B demonstrates that IGM and its regularized variants achieve higher correlations between measured and predicted fluxes across conditions compared to all single-condition methods, including E-flux2. This ability to capture dynamic flux changes is crucial for studying metabolic adaptations and regulatory mechanisms. Unlike single-condition methods, IGM utilizes cross-condition information, providing a holistic and consistent framework for comparative metabolic studies.

Overall, IGM and its extended versions (IGM + L1 and IGM + L2) outperform single-condition methods in both within-condition accuracy and cross-condition dynamic consistency. Among these, IGM + L1 consistently delivers the highest performance across both metrics (Fig 3A and 3B), demonstrating the benefits of incorporating L1 regularization to balance prediction accuracy and flux dynamic fidelity. These results highlight IGM’s effectiveness as a multi-condition integration framework, making it particularly suitable for comparative metabolic studies and dynamic pathway analyses.

### Analysis of biomass objective coefficient and gene expression transformation functions in IGM

**Fig 4:**
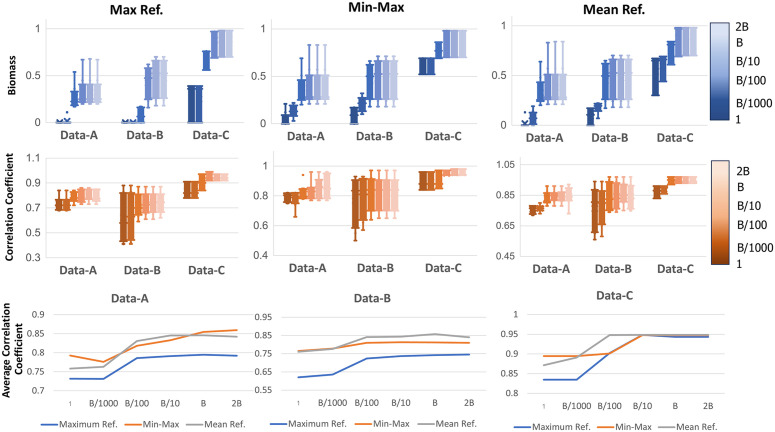
The impact of varying the objective coefficient of biomass reaction flux and different gene expression transformation functions in Data-A, Data-B, and Data-C. The first and second rows present boxplots of the predicted biomass levels and the correlation coefficients between predicted and measured fluxes, respectively. These predictions are obtained using IGM with different gene expression transformation functions: maximum gene expression as a reference, min-max scaling, and average gene expression as a reference. Each method is evaluated across various objective coefficient values for biomass flux: 1, *B*/1000, *B*/100, *B*/10, *B*, and 2*B*. The third row displays line plots of the average correlation coefficient in Data-A, Data-B, and Data-C, respectively, illustrating how changes in the biomass objective coefficient affect predictive performance. Each subplot corresponds to a different dataset, showing the trends across the three gene expression transformation methods. There are three lines which are blue, orange, and grey color which represent using maximum gene expression as reference, max-min scaling, and average of gene expression as reference, respectively.

Integrating gene expression data into metabolic models involves multiple parameters and transformation functions, each of which can significantly influence model predictions. In this study, we focus on two key factors: (i) the coefficient of the biomass reaction flux ($\tilde{c}$), and (ii) the function used to transform gene expression levels, *f*(*g*). The effects of varying these parameters and functions are illustrated in Fig 4. We analyze predicted biomass levels and correlation coefficients between predicted fluxes and measured fluxes under different parameter settings. The biomass objective coefficient is varied across six values: 1, *B*/1000, *B*/100, *B*/10, *B*, and 2*B*, where *B* is a constant defined as $(\text{2}k+\text{2}n)/v_{biomass}^{max}$. Here, $v_{biomass}^{max}$ is the maximum biomass flux obtained from FBA by maximizing biomass reaction. The rationale for defining B in this way is based on its role as the normalized weight of the biomass term in the IGM objective function. Specifically, it is divided by $v_{biomass}^{max}$ which produces a value between 0 and 1, making it comparable in scale to other variables in objective function. It is then multiplied by the maximum possible value of the nonnegative variables in the IGM objective function, which equals 2k+2n. This normalization ensures that the model can simultaneously maximize biomass production and minimize deviations between relative gene expression and flux values, while keeping the biomass term in comparable magnitude to other components in the objective.

For gene expression data, we consider three transformation functions: (i) using the maximum of gene expression value as a reference, (ii) min-max scaling, and (iii) using the mean gene expression value as a reference, as described in Eqs (7)–(10). Fig 4 is structured into three rows, each highlighting different aspects of the model’s behavior. The first row presents predicted biomass levels, the second row shows the correlation coefficients between predicted and measured fluxes, and the third row provides a comparative analysis of transformation functions based on their predictive performance.

The first row of Fig 4 displays boxplots for predicted biomass levels across datasets (Data-A, Data-B, and Data-C) for each transformation function. These results show how varying the biomass flux coefficient in IGM affects biomass predictions. When the biomass flux coefficient is set to a small negative value, the predicted biomass level approaches zero across all transformation functions. As the coefficient becomes more negative, biomass production increases, consistent with the mathematical formulation of IGM, where minimizing a negative biomass flux is equivalent to maximizing biomass production.

To assess prediction accuracy, we examine the correlation coefficients between predicted and experimentally measured fluxes, as shown in the second row of Fig 4. Across all datasets and transformation functions, increasing the biomass objective coefficient improves correlation coefficients, indicating enhanced prediction accuracy. These findings highlight that selecting an appropriate biomass coefficient is crucial for balancing biomass optimization with gene expression constraints. In contrast, using a small negative coefficient for the biomass term (i.e., placing low emphasis on biomass maximization) severely degrades model performance and leads to poor agreement between predicted and measured fluxes. Increasing the magnitude of this negative coefficient (i.e., placing stronger emphasis on biomass maximization) improves the prediction accuracy. However, removing the gene expression term entirely—equivalent to using only the biomass objective as in traditional FBA—also results in lower correlation coefficients, as shown in Fig 2A. These observations highlight the need for a balanced, hybrid objective function that integrates both biomass optimization and gene expression information*.*

The third row of Fig 4 presents line plots comparing the three transformation functions based on average correlation coefficients across Data-A, Data-B, and Data-C. These subplots visualize how varying the biomass objective coefficient influences predictive performance under different transformation functions. To focus on the most biologically relevant parameter ranges, we primarily examine cases where the biomass coefficient is greater than or equal to B/10, as this range has demonstrated better performance in the first and second rows of Fig 4.

Overall, while the three transformation functions produce comparable performance, their effectiveness varies depending on the experimental context of each dataset. In Data-A (genetic and environmental perturbations in *E. coli*), max-min scaling performs best. This is likely due to the structured nature of the perturbations (e.g., single-gene deletions, varying dilution rates), which induce significant and controlled shifts in gene expression. Max-min scaling captures these wide expression ranges effectively, aligning well with the flux data derived from ^13^C-labeled experiments. In contrast, Data-B, which involves growth on diverse carbon sources, shows a marked performance drop with max-based transformation. This dataset introduces heterogeneous nutrient environments that likely lead to variable expression ranges and potential outliers. As a result, the mean-based transformation outperforms others, offering a more stable reference point less sensitive to extreme values. Similarly, Data-C, profiling strain-specific responses (e.g., NADH oxidase and ATPase mutants), also favors the mean-based transformation. Here, expression changes are subtler and strain-specific, so using the mean helps reflect consistent expression shifts while avoiding distortion from high-expression genes.

Collectively, these results suggest that mean-based transformation offers the most robust and generalizable approach for integrating gene expression data into metabolic models. While max-min scaling remains effective for well-controlled perturbation studies like Data-A, its advantage over mean-based transformation is minimal in most cases.

### Concordance between relative gene expression and model-derived expression variables via IGM

**Fig 5:**
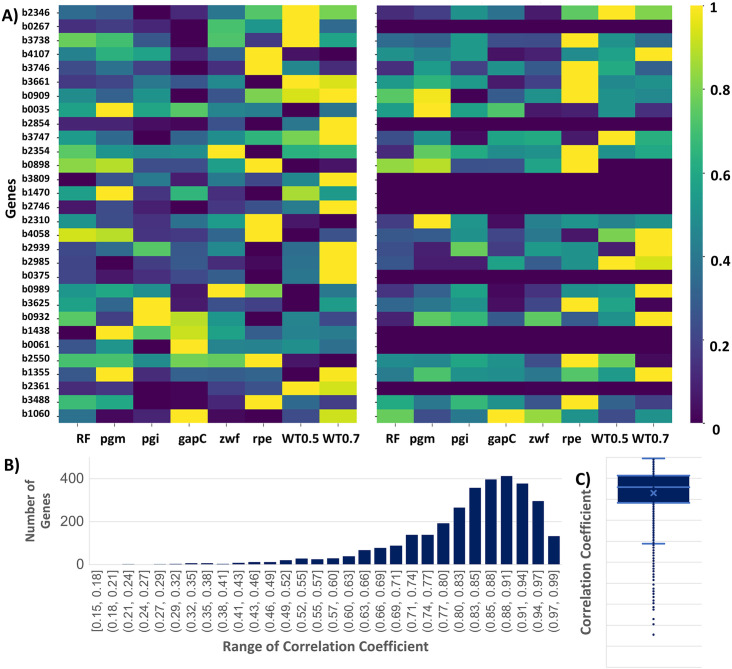
Relative gene expression values and gene expression variable values of Data-A. **(A)** Heatmap showing 30 randomly selected relative gene expression profiles (left panel) and gene expression variable values in IGM programming (right panel) across eight conditions: reference (RF), WT0.2 (wild type at 0.2 per hour), WT0.5 (wild type at 0.5 per hour), WT0.7 (wild type at 0.7 per hour), and single gene deletions (*pgm*, *pgi*, *gapC*, *zwf*, and *rpe*). The heatmap values range from 0 to 1, with blue indicating values near 0 and yellow indicating values near 1. **(B)** Histogram showing the distribution of correlation coefficients between relative gene expression values and gene expression variable values. **(C)** Box plot of correlation coefficients between relative gene expression values and gene expression variable values.

IGM achieved close alignment between relative gene expression inputs and optimized gene expression variables, enabling more accurate integration of transcriptomic data into metabolic flux predictions. To demonstrate the quality of this integration, the heatmap plots for the relative gene expression inputs and the optimized gene expression were compared. Results for Data-A are shown here in Fig 5, while those for Data-B and Data-C are provided in S3 and S4 Figs within S1 File. For each dataset, 30 genes were randomly selected with their relative gene expression values (left panel in Fig 5A) and optimized gene expression variable values (right panel in Fig 5A) compared across eight conditions, including three wild-type growth rates (WT0.2, WT0.5, WT0.7) and five perturbations involving single gene deletions (*pgm*, *pgi*, *gapC*, *zwf*, and *rpe*). In most cases, the optimized gene expression variables closely matched the input patterns, demonstrating that IGM programming effectively preserves the underlying biological trends.

However, a subset of genes (e.g., *b0267*, *b2854*, *b3809*, *b1470*, *b2746*, *b0375*, *b1438*, *b0061*, and *b2361*) exhibited zero-valued gene expression variables across all conditions. This suggests that while these genes are transcribed (non-zero expression), they do not contribute to any active reactions in the metabolic network. These genes may encode non-essential enzymes under the tested conditions or may be involved in pathways that are inactive or disconnected from the central metabolism. This highlights an important feature of IGM that its capacity to filter out non-contributing genes, allowing more reliable mapping from transcriptome to flux-related variables.

To quantitatively evaluate this consistency across the entire dataset, we computed the correlation coefficients between the relative gene expression values and the corresponding gene expression variable values for each gene. The distribution of these correlations (Fig 5B) is strongly skewed toward high positive values, and the box plot (Fig 5C) confirms that the majority of genes have correlation coefficients above 0.8. The average correlation across all genes is approximately 0.86, with most genes falling within the 0.88–0.91 range in both datasets. Similar results for Data-B supporting the same conclusion, are provided in S3 Fig within S1 File. For Data-C, the results are also presented in S4 Fig within S1 File. Since Data-C contains only three conditions, the correlation coefficients are notably high. The average correlation across all genes is approximately 0.91, with most genes falling within the 0.95–0.98 range. This high correlation reflects the robustness of IGM in integrating transcriptomic data.

Importantly, this high level of correlation suggests that IGM programming successfully bridges the gap between gene expression and metabolic activity, overcoming a common challenge in constraint-based modeling. These results support the hypothesis that incorporating gene-level information can enhance the predictive capacity of genome-scale metabolic models, especially under diverse physiological states. Furthermore, this framework can serve as a foundational tool for condition-specific metabolic modeling.

### Flux change analysis

Using the IGM framework, we calculated the reaction flux distributions constrained by gene expression data. These flux solutions were consistent with each experimental condition.

To evaluate changes in metabolic activity across conditions, we performed flux change analysis for each dataset. All heatmaps of the normalized average flux values in each subsystem across conditions for each dataset are provided in S5 Text in S1 File (S5A, S6A, and S7A Figs for Data-A, Data-B, and Data-C, respectively). We further compare the encoded gene variables through GPR rule by plotting the heatmap of average encoded gene variable values in each subsystem across conditions. (S5B-S7B Figs). These heatmaps show the consistence between flux and encoded gene variable values. However, the flux values in Data-B are more variate than Data-A, and Data-C. For depth study, we thus show the scatter plots of flux changes for Data-A, Data-B, and Data-C are presented in S8-S10 Figs (S6 Text in S1 File), respectively. Each figure contains subplots comparing reaction fluxes (in log scale) between every pair of conditions, with the dashed line *y* = *x* representing identical flux values in the two conditions. These analyses demonstrate that both genetic perturbations and changes in carbon source significantly influence reaction flux distributions. Importantly, the comparison across wild-type growth rates indicates that higher growth rates are associated with globally increased fluxes, while specific gene knockouts can selectively elevate flux through particular pathways, suggesting compensatory metabolic responses.

**Fig 6:**
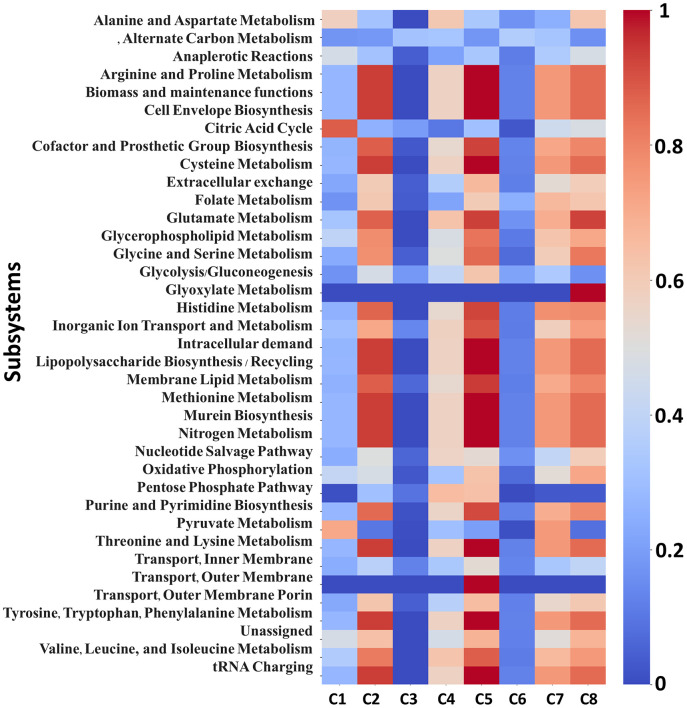
Flux solution change analysis for Data-B. Heatmap showing the row-normalized average flux values in each metabolic subsystem across conditions C1 to C8, representing eight carbon sources: acetate, fructose, galactose, gluconate, glucose, glycerol, pyruvate, and succinate, respectively. The heatmap values range from 0 to 1, with blue tone indicating values near 0 and red tone indicating values near 1.

Among these, the heatmap and scatter plot for Data-B shows more pronounced variations than the other datasets; therefore, we focus on its results here to further confirm the performance of IGM. Fig 6 presents the heatmap of normalized average flux values across eight conditions (C1–C8), each corresponding to a distinct carbon source: acetate, fructose, galactose, gluconate, glucose, glycerol, pyruvate, and succinate, respectively. Notably, conditions C2 and C5 (fructose and glucose) exhibit higher overall flux activity across many subsystems compared to other carbon sources, whereas C3 and C6 (galactose and glycerol) show markedly lower flux distributions in multiple subsystems.

**Fig 7:**
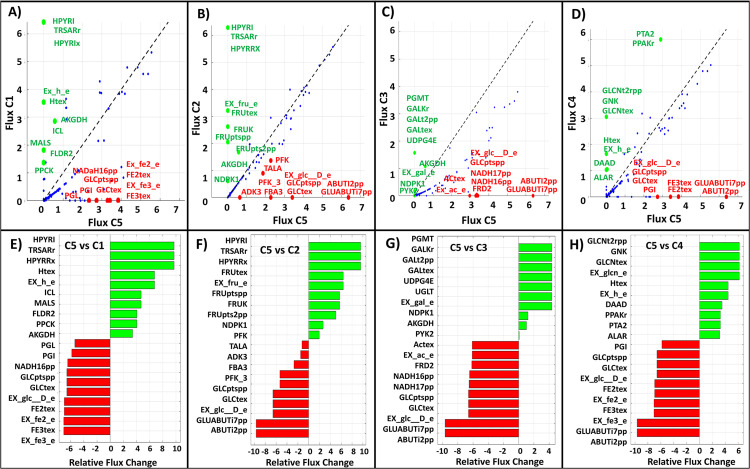
(A – D) Scatter plots of reaction flux changes comparing the glucose carbon source condition (C5) with four other conditions: acetate (C1, Fig 7A), fructose (C2, Fig 7B), galactose (C3, Fig 7C) and gluconate (C4, Fig 7D). The *x*-axis represents the flux values (log scale) under the glucose condition, and the y-axis represents the flux values (log-scale) under the other conditions. The black dashed line indicates line *y* = *x*. The green and red nodes highlight the top 10 upregulated and downregulated relative reaction fluxes, respectively, with reaction names labeled; all other reactions are shown as blue nodes. **(E – H)** Horizontal bar plots of the top 10 upregulated (green) and downregulated (red) reactions, along with their relative flux scores, for the same comparisons: C5 vs. C1 (Fig 7E), C5 vs. C2 (Fig 7F), C5 vs. C3 (Fig 7G), and C5 vs. C4 (Fig 7H).

To further investigate these differences, we can visualize the flux change analysis via IGM framework to identify the top 10 most up- and down-regulated reactions between C5 (control) which use glucose as the carbon sources, and other seven conditions (threat), which use different carbon sources: C1–C4 (Fig 7) and C6–C8 (S11 Fig in S1 File) in Data-B, based on the log2-transformed relative flux changes. In Fig 7 (panels A, B, C, and D) and S11 Fig (panels A, B, and E) in S1 File, scatter plots show reaction flux changes comparing the glucose carbon source condition (C5) with the other seven conditions. The black dashed line indicates y = x, representing identical flux values in both conditions. Green and red nodes highlight the top 10 upregulated and downregulated relative reaction fluxes, respectively, with reaction names labeled. These values were calculated as the logarithm of the ratio between fluxes in the threat condition and fluxes in the control condition. We found that fluxes related to each specific carbon source pathway were consistently among the top 10 upregulated or downregulated relative reaction fluxes. Furthermore, Fig 7 (panels E, F, G, and H) and S11 Fig (panels C, D, and F) in S1 File present horizontal bar plots of the top 10 upregulated (green) and downregulated (red) reactions, along with their relative flux scores. These visualizations highlight the metabolic reactions most affected by changes in the carbon source, both in terms of absolute flux magnitude and relative variation.

These results demonstrate that the IGM framework effectively captures condition-specific metabolic adaptations by adjusting the proportional distribution of reaction fluxes in a manner consistent with the supplied gene expression data. The observed pathway-specific regulation confirms the model’s ability to reflect biologically meaningful shifts in metabolism driven by different carbon sources.

## Discussion

Genome-scale metabolic models (GEMs) have become indispensable tools for investigating cellular metabolism across a wide range of organisms. Their ability to integrate biochemical, genetic, and physiological data enables detailed simulation and analysis of metabolic processes under various environmental and genetic conditions. Among the most widely used analytical approaches is Flux Balance Analysis (FBA), which despite its utility relies heavily on predefined constraints and objective functions. This often results in multiple equally optimal flux solutions that differ in biological plausibility, particularly under heterogeneous or dynamic conditions. To address these limitations, several methods have been developed to integrate transcriptomic data into GEMs. These techniques are typically categorized into two main types: those that use gene expression data from a single condition and those that use data from multiple conditions to adjust flux constraints or objectives. While useful, many of these methods depend on arbitrary thresholds, fail to preserve flux units, or require extensive prior knowledge. Some also struggle with limited generalizability or high data requirements. In particular, single-condition-based approaches are less effective for comparative or dynamic studies.

In light of these challenges, we propose the IGM framework, which builds upon and extends existing methodologies by enabling the consistent integration of relative gene expression data across multiple conditions within a mixed-integer linear programming (MILP) formulation. IGM requires three main inputs: gene expression data from multiple conditions, a genome-scale metabolic model (GEM), and a set of initial carbon sources. The framework consists of three key steps. First, flux balance analysis (FBA) is applied using the specified carbon sources to predict the maximum biomass production. Next, flux variability analysis (FVA) is performed to determine the possible minimum and maximum flux values for each reaction. Finally, the results from FVA and the relative gene expression data are incorporated into the IGM model. The objective of IGM is to maximize biomass production while minimizing the inconsistency between relative gene expression and corresponding relative flux values. To compute relative gene expression, we propose three transformation techniques: using the maximum expression across conditions as a reference, applying min–max scaling, and using the mean expression as a reference. In addition, we introduce a method for mapping gene expression data to the metabolic model, which facilitates the implementation of gene-reaction relationship constraints. To further enhance robustness, we extend IGM with norm regularization. By avoiding binarization and preserving flux units, the IGM framework and its norm-regularized variants provide a more interpretable and biologically consistent representation of condition-specific metabolism.

Evaluations on the *E. coli* model across three datasets demonstrated improved correlation with experimentally measured fluxes, suggesting that our approach effectively captures meaningful metabolic shifts while maintaining computational efficiency. Compared to traditional FBA and single-condition integration methods, IGM offers a more robust platform for studying metabolism under diverse biological contexts.

Our proposed method, IGM, demonstrates superior predictive performance across multiple evaluation criteria and datasets. IGM improves flux prediction by incorporating gene expression data, significantly outperforming traditional FBA in terms of the correlation between predicted and experimentally measured fluxes. This integration enhances both the biological relevance and accuracy of flux predictions. Analysis of reaction flux flexibility reveals that IGM typically results in a smaller feasible solution space than FBA, contributing to increased specificity and reduced ambiguity in flux predictions. This finding further supports the conclusion that incorporating gene expression data effectively constrains the model toward more biologically meaningful solutions. The extended versions of IGM, namely IGM + L1 and IGM + L2, further improve performance through the introduction of regularization. Among these, IGM + L1 achieves the best results, with the highest correlation coefficients and lowest normalized root mean square error (NRMSE) values, while also reducing prediction variability. This indicates that L1 regularization not only enhances accuracy but also stabilizes model predictions across diverse conditions. Compared to established single-condition methods such as GIMME, E-flux, and E-flux2, IGM-based methods consistently perform better, particularly in capturing dynamic changes while maintaining predictive consistency across multiple conditions. This underscores IGM’s suitability for comparative and dynamic metabolic studies.

However, the performance of the model is sensitive to the choice of biomass objective coefficient and the gene expression transformation function. Results indicate that a larger (i.e., more negative) biomass coefficient generally enhances prediction accuracy. Among the transformation functions evaluated, using the mean of gene expression values provides the most robust and consistent performance across datasets. Despite these sensitivities, IGM offers a novel framework for capturing the dynamic behavior of reaction fluxes, yielding new insights into previously uncharacterized aspects of cellular metabolism.

In our study, we also assessed the performance of this integration by comparing relative gene expression values with their corresponding model-derived expression variables across multiple conditions. The visual concordance between the original gene expression profiles and the values generated by IGM indicates that the model preserves the core patterns of the transcriptomic input. This alignment confirms that the optimization process effectively maintains cross-condition consistency and biological relevance while respecting network constraints. Interestingly, we observed a group of genes whose expression variables were uniformly zero across all conditions, despite having non-zero transcript levels. This observation implies that while these genes are transcribed, they do not contribute to flux-carrying reactions in the model. These genes may encode enzymes that are non-essential in the tested environments or associated with inactive pathways. This filtering effect highlights the utility of IGM in distinguishing between transcriptional noise and functionally relevant expression, a limitation often encountered in traditional expression-constrained modeling. To quantitatively assess model consistency at the genome scale, we computed Pearson correlation coefficients between the relative gene expression values and gene expression variable values for each gene. The distribution of correlation coefficients was skewed toward high positive values, with most genes exhibiting correlations above 0.8. The average correlation across all genes reinforces the conclusion that IGM integrates transcriptomic data while preserving system-level constraints.

Moreover, we demonstrated the application of IGM for estimating biologically relevant reaction fluxes constrained by gene expression data across multiple environmental conditions. Through flux change analysis, we identified distinct metabolic shifts associated with different conditions.

Our results revealed that flux regulation patterns aligned with known substrate-specific pathways, validating the model’s ability to represent condition-dependent metabolic behavior. This confirms the utility of IGM in uncovering regulatory shifts in metabolism and supports its broader applicability in systems biology and metabolic engineering studies.

## Conclusion

In this study, we introduced IGM: Integrated Gene-expression Modeling a novel MILP-based framework for the consistent integration of relative gene expression data across multiple conditions into genome-scale metabolic models. By incorporating flux variability analysis and gene expression-derived constraints without binarization, IGM enhances the biological interpretability and predictive accuracy of flux distributions. Our results on *E. coli* models demonstrate that IGM significantly improves correlation with experimentally measured fluxes, reduces flux ambiguity, and offers more consistent predictions across dynamic conditions. The extended versions, IGM + L1 and IGM + L2, further enhance performance through regularization, with IGM + L1 yielding the most robust results. Comparative analyses highlight that IGM outperforms traditional FBA and widely used single-condition integration methods, establishing its suitability for dynamic, comparative, and multi-condition metabolic studies. Although model performance depends on factors such as biomass objective coefficients and gene expression transformation functions, our findings suggest that IGM provides a powerful and flexible platform for advancing the study of consistent condition-specific metabolism and uncovering dynamic metabolic adaptations.

## Supporting information

S1 File Supporting Information. This file contains supplementary methods and additional results including supplementary texts (S1–S6) and figures (S1–S11) referenced in the main manuscript.
(PDF)

S2 File Details of the data used in this study. This file contains all input datasets used for the IGM analysis, including gene expression data, uptake reactions, measured fluxes, and the genome-scale metabolic model.
(XLSX)
