# Supplementary material for: IGM: Integrated Gene-expression Modeling for multi-condition flux-preserving genome-scale metabolic models
Source: PLoS One. 2026 Feb 9;21(2):e0342294. doi: 10.1371/journal.pone.0342294 (PMC12885322; doi:10.1371/journal.pone.0342294)
Supplement: S1 File — This file contains supplementary methods and additional results including supplementary texts (S1–S6) and figures (S1–S11) referenced in the main manuscript. (PDF) [file pone.0342294.s001.pdf]

Supplementary Information

S1 Text. Gene Expression Transformation and Its Implications for GPR-Based Reaction Scoring

Our framework normalizes gene expression values before calculating reaction scores under the GPR rules. However, many studies compute reaction scores directly from raw gene expression using the GPR rules and apply normalization only afterward. This classical approach preserves absolute expression magnitude and makes the resulting reaction score independent of the sample size, which is particularly useful for large datasets such as single-cell data. In contrast, our model normalizes gene expressions prior to computing reaction scores. This choice is motivated by the fact that the optimization framework does not use absolute gene expression, but relative activity measures. Normalizing first allows each gene to be interpreted on a [0, 1] scale, reducing the impact of extreme values and enabling a more comparable representation of transcriptional activity across conditions. Importantly, this prevents highly expressed genes from dominating the GPR mapping simply due to scale differences, and ensures that the min/max logic reflects biological regulatory structure rather than raw magnitude. Under this formulation, the GPR-derived quantity $\hat{g}_j$ represents the relative metabolic potential of a reaction, which is more consistent with the model's objective of integrating gene expression into flux predictions (see the formulation in the main text).

To clarify the conceptual implications of this classical approach and to explain the rationale behind normalizing gene expression prior to computing the reaction scores, the example below provides a more detailed illustration.

In the standard pipeline of the classical approach, GPR-based scores are computed first and normalization is applied afterward. Raw gene expression values are initially aggregated using the GPR rules—min for AND relationships and max or sum for OR relationships—after which the resulting reaction scores are normalized at a later stage. To illustrate this process, consider the following expression values.

	C1	C2	C3	C4
Gene1	5	7	7	15
Gene2	2	10	8	4

For a reaction with the rule R1 = (g_1 OR g_2). Applying *Sum approach*, R1 = (g_1 + g_2), we obtain

Condition	C1	C2	C3	C4
R1	7	17	15	19

Applying *Max approach*, R1 = max(g_1, g_2), we obtain

Condition	C1	C2	C3	C4
R1	5	10	8	15

This approach preserves *absolute* expression magnitudes and does not depend on how the data are scaled across samples. One advantage is that the reaction score remains formally independent of the number of biological samples. However, such scores can be dominated by highly expressed genes or technical variability between samples, which may limit their ability to reflect *relative functional activity* across conditions.

In contrast, our method normalizes gene expression *across conditions before* applying the GPR rules. Conceptually, this extends the notion of binary gene activity (0/1) into a continuous scale between 0 and 1, enabling a more interpretable comparison of relative transcriptional activity across conditions. For example:

Using max normalization, we obtain

	C1	C2	C3	C4
$\tilde{g}_1$	0.33	0.47	0.47	1.00
$\tilde{g}_2$	0.20	1.00	0.80	0.40

Applying the rule R1 = (g_1 OR g_2), we obtain

Condition	C1	C2	C3	C4
R1	0.33	1.00	0.80	1.00

Under this representation, both C2 and C4 show high reaction activity, but for different biological reasons: g_2 drives activity in C2, whereas g_1 drives activity in C4. The method therefore highlights *relative functional activity*, which is the central focus of our study.

The reason why we need to normalize the gene expression values first is to address extreme values and comparability across conditions. Some genes exhibit very high or very low expression, which can disproportionately influence reaction scores if raw values are directly aggregated. For comparability across experimental conditions, the normalized values represent *relative activity* within each gene across conditions, reducing confounding effects from differences in sequencing depth or dynamic range. Furthermore, normalized values map naturally onto a [0,1] scale, facilitating direct interpretation as relative levels of potential metabolic activity. This also supports integration with downstream optimization. Since our optimization framework requires relative activity measures rather than absolute magnitudes, normalization prior to GPR computation produces quantities more aligned with the model assumptions.

Different normalization strategies can preserve different aspects of the gene expression structure. For example, using min–max scaling on the entire array as this formulation

$$\tilde{g}_{i,j} = \frac{T_{i,j} - \min(T_i)}{\max(T_i) - \min(T_i)}.$$

We obtain

	C1	C2	C3	C4
$\tilde{g}_1$	0.23	0.38	0.38	1.00
$\tilde{g}_2$	0.00	0.61	0.46	0.15

After applying the GPR rule, it results in

Condition	C1	C2	C3	C4
R1	0.23	0.61	0.46	1.00

This transformation preserves the global expression structure of each gene—i.e., the relative differences in magnitude among genes—while mapping values to a [0,1], enabling flexibility in choosing normalization functions depending on the application. Selecting an appropriate normalization scheme remains a methodological consideration, as different transformations (e.g., max normalization, z-score scaling, quantile normalization) emphasize different aspects of the data.

Normalizing gene expressions prior to score computation includes certain limitations. Scores become sensitive to the selected normalization method and, to some extent, to sample size, as score distributions may shift when the number of samples increases. Moreover, some absolute expression information is partially lost. Nonetheless, because our method evaluates relative transcriptional activity across conditions and incorporates these values as soft constraints within the optimization framework, pre-normalization yields inputs that are more stable, interpretable, and aligned with the modeling objectives of this study. Normalization also prevents highly expressed genes from disproportionately dominating the reaction score due to technical or experimental variability, and ensures that all genes are compared on a common scale consistent with the underlying optimization model.

S2 Text. Formulation for function of gene-protein-reaction association

In our IGM programming framework, we transform gene-protein-reaction (GPR) rules from a max–min function representation into mixed-integer constraints. This allows us to handle the logical structure of GPRs directly within a mixed-integer linear programming (MILP) formulation. The example of a GPR association is illustrated in Figure S1. GPR associations capture the connections between genes, proteins, and metabolic reactions through Boolean logic, formulated using "AND" and "OR" operators. An "AND" relationship in a GPR rule indicates that an enzyme complex is composed of multiple gene products, requiring all subunits to be present for the reaction to carry flux. In contrast, an "OR" relationship denotes the presence of isozymes—distinct enzymes encoded by different genes that can each catalyze the same reaction.

In the manuscript, Equations (19)-(21) describe the mixed-integer constraints for the case of an 'AND' relationship, while Equations (22)-(24) describe the constraints for an 'OR' relationship. However, in practice, GPR rules are often more complex — they may involve more than two genes and a mixture of 'AND' and 'OR' relationships.

Below, we present a step-by-step procedure for transforming GPR rules from the simplest to the most complex form.

Let:

- $\hat{g}_j$ = the effective gene expression for reaction j, determined through the GPR function.
- g_i = the relative gene expression of gene iii.
- y_i = binary variable (0 or 1) used for logical encoding.
- M = a sufficiently large positive constant ("big-M" parameter).
- R_j = the set of genes associated with reaction j.

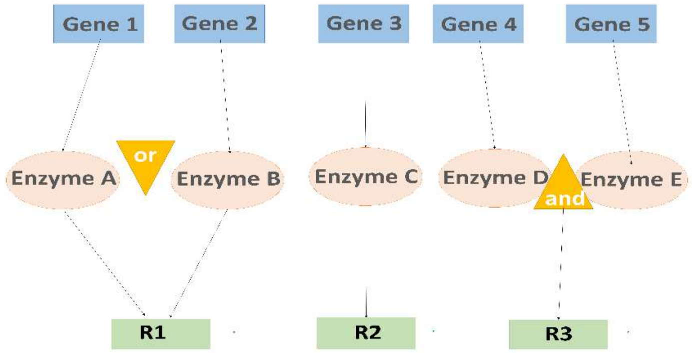

S1 Fig. Example of Gene-protein-reaction (GPR) association

Case 1 If only one gene is associated with a reaction, the effective gene expression is simply:

$$\hat{g}_j = g_i$$

This case does not require any integer variables.

Case 2 Multiple genes (k genes) in an 'AND' relationship

If a reaction requires all k genes in R_j to be present ('AND' relationship), the effective expression is constrained by the smallest expression level among them.

$$\hat{g}_j \leq g_i \qquad \text{for all } i = 1,2,3,\dots,k \text{ in } R_j \text{ relationship;}$$
$$\hat{g}_j \geq g_i - My_i \qquad \text{for all } i = 1,2,3,\dots,k \text{ in } R_j \text{ relationship;}$$
$$\textstyle\sum_{i=1}^{k} y_i = k - 1,$$

Case 3 Multiple genes (k genes) in an 'OR' relationship

If a reaction is active when any of k genes is present ('OR' relationship), the effective expression is constrained by the largest expression level among them.

$$\hat{g}_j \geq g_i \quad \text{for all } i = 1,2,3,\dots,k \text{ in } R_j \text{ relationship;}$$
$$\hat{g}_j \leq g_i + My_i \quad \text{for all } i = 1,2,3,\dots,k \text{ in } R_j \text{ relationship;}$$
$$\sum_{i=1}^{k} y_i = k-1,$$

Case 4 Combination of 'AND' and 'OR' relationship

In more complex cases, a single GPR rule may include both 'AND' and 'OR' operators. We handle this by splitting the GPR rule by 'OR' first, and then processing each resulting component separately.

Let c be the number of components after splitting by 'OR'. We introduce c new variables: q_1, q_2, q_3, ..., and q_c, each representing the effective expression of one 'OR' component.

- For each component:
  - If it contains multiple genes in an 'AND' relationship, apply **Case 2** to compute m_k.
  - If it contains only a single gene, apply **Case 1**.
- After all components are computed, combine them using **Case 3** to form the final $\hat{g}_j$.

For example: $\hat{g}_1$: (g1 and g2) or (g3 and g4 and g5) or (g6)

This GPR is split by 'OR', resulting in three components: (g1 and g2), (g3 and g4 and g5), and (g6). Therefore, we introduce three variables for encoding which are q_1, q_2, and q_3. We consider 'AND' in each component. In first component, there is 'AND', then we can transform this by using Case 2, as follows:

$$q_1 \leq g_1$$
$$q_1 \leq g_2$$
$$q_1 \geq g_1 - My_1$$
$$m_1 \geq g_2 - My_2$$
$$y_1 + y_2 = 1$$

In the second component, there are three genes in 'AND' relationship. Therefore, we obtain

$$q_2 \leq g_3$$
$$q_2 \leq g_4$$
$$q_2 \leq g_5$$
$$q_2 \geq g_3 - My_3$$
$$q_2 \geq g_4 - My_4$$
$$q_2 \geq g_5 - My_5$$
$$y_3 + y_4 + y_5 = 2$$

In the last component, there is no 'AND'. Therefore, $q_3 = g_6$.

In the last step, we combine these three components by 'OR'. Thus, we obtain

$$\hat{g}_1 \geq q_1$$
$$\hat{g}_1 \geq q_2$$
$$\hat{g}_1 \geq q_3$$
$$\hat{g}_1 \leq q_1 + My_6$$
$$\hat{g}_1 \leq q_2 + My_7$$
$$\hat{g}_1 \leq q_3 + My_8$$
$$y_6 + y_7 + y_8 = 2.$$

In our framework, the score assigned to each reaction (denoted as $\hat{g}_j$) is computed based on the GPR rules and the corresponding relative gene expression levels. Although this score could be calculated beforehand as a fixed deterministic value, we opted to embed it directly within the optimization problem for two reasons.

- Dynamic adjustment: Integrating $\hat{g}_j$ into the optimization allows the model to adaptively modulate the influence of gene expression on reaction fluxes across different conditions or constraints. This is especially crucial when genes participate in multiple reactions or are shared among reactions, as the optimization maintains network-wide consistency.
- Flexibility for future extensions: Including $\hat{g}_j$ within the optimization also provides the framework with the flexibility to incorporate additional constraints reflecting gene-gene relationships. This design renders the model more versatile and extensible, facilitating the future integration of constraints derived from regulatory or co-expression networks.

S3 Text. Matrix Formulation of the IGM Optimization Problem

In the manuscript, we presented the optimization formulation of IGM in its standard equation form. This formulation can also be rewritten in matrix form, which corresponds directly to the structure implemented in the programming code. The matrix formulation is described below.

We solve the following optimization problem:

$$\begin{aligned} & Minimize\ c^T x \\ & \text{Subject to} \quad Aeq * x = Beq \\ & \qquad\qquad\quad A * x \leq B \\ & \qquad\qquad\quad lb \leq x \leq ub \end{aligned}$$

Decision variable vector

The decision variable vector is: $x = [v^T, g^T, p^T, \delta^T, \varepsilon^T, y^T, b^T]^T$

Where:

- v = vector of reaction fluxes.
- g = vector of gene variables.
- q = vector of new variables encoding the gene expression through the GPR rule for each reaction flux (constructed in the case of complicated GPRs, as described in Section S1 above).
- δ = difference between the measured relative gene expression and the corresponding gene variables, with $\delta = \{\delta_i^1, \delta_i^2\}$ for $i = 1,2,3, \ldots, k$.
- ε = difference between the relative gene expression value (through the GPR rule) and the relative fluxes, with $\varepsilon = \{\varepsilon_i^1, \varepsilon_i^2\}$ for $i = 1,2,3, \ldots, n$.
- y = vector of binary variables defined in the GPR rule formulation (used to model min/max logic).
- b = vector of binary control variables for irreversible reaction handling in the metabolic model.

Objective function coefficients

The coefficient vector c has:

- All elements set to 0, except:
  - The coefficient for the biomass reaction is $-\tilde{c}$ (negative for maximization in a minimization framework).
  - The coefficients for all elements of δ and ε are 1 (to penalize deviations in gene and flux expression).

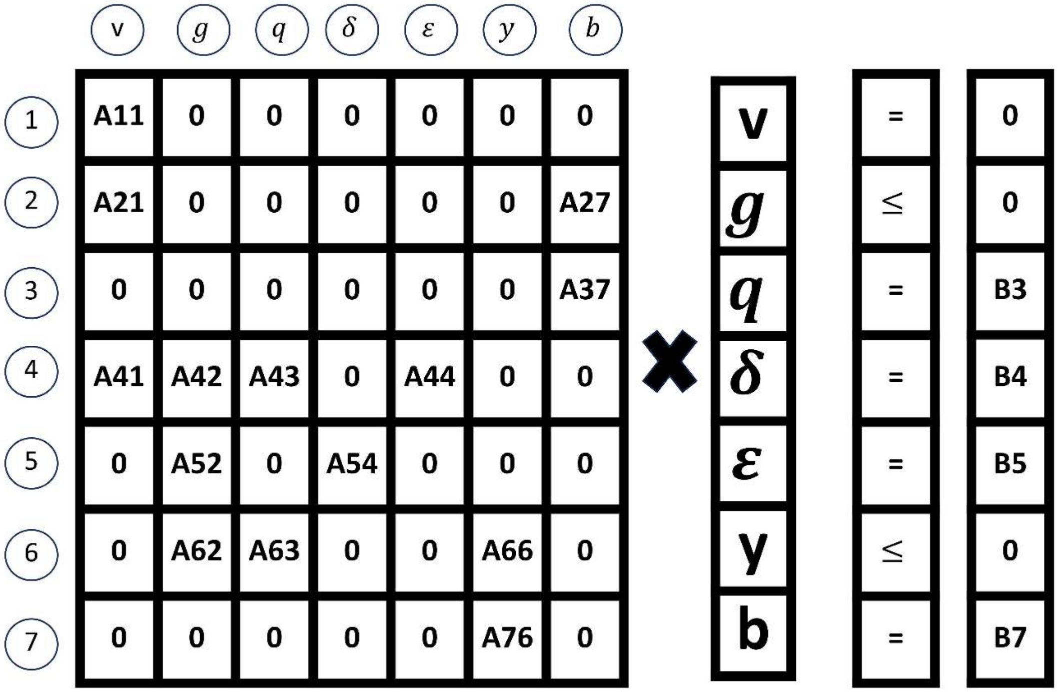

S2 Fig. The representation matrix of IGM constraint for equality and inequality in optimization problem.

Matrix block structure

The inequality matrix and vector are:

$$A = \begin{bmatrix} A_2 \\ A_7 \end{bmatrix}, \qquad B = [B_2^T, B_7^T]^T$$

Each block A_i is partitioned into column blocks:

$$A_i = [A_{i,1}, \qquad A_{i,2}, \dots, A_{i,7}]$$

where $A_{i,j}$ is the submatrix corresponding to the variables in column j of x, and B_i is the corresponding RHS vector.

The equality constraint matrix $Aeq = \begin{bmatrix} A_1 \\ A_3 \\ A_4 \\ A_5 \\ A_6 \end{bmatrix}$,

and the RHS vector $Beq = [B_1^T, B_3^T, B_4^T, B_5^T, B_6^T]^T$ (as shown in Fig. S2). Vectors lb and ub define the lower and upper bounds of all variables.

Description of submatrices

Below we detail the meaning of each submatrix in Fig. S2.

Row 1 – Steady-state constraint

The first block ($A_{1,1}$) enforces the steady-state condition:

$$A_{1,1}v = 0$$

Here, $A_{1,1} = S$, where S is the stoichiometric matrix of the metabolic model.

Row 2 – Flux upper bound control (irreversible reaction handling)

The second block sets the upper bound on reaction fluxes using binary variables b:

$$0 \leq v \leq U^T b$$

Which can be rewritten as: $v - U^T b \leq 0$

Thus: $A_{2,1} = I_{n\times n}$, $A_{2,7} = -U^T \times I_{n\times n}$

forming: $A_{2,1}v + A_{2,7}b \leq 0$

Row 3 – Reversible reaction decomposition

For reversible reactions, we require that only one direction is active:

$$b_i + b_j = 1 \quad \text{for all } (i,j) \in RE$$

Here, RE is the set of forward/reverse reaction index pairs from the same reversible reaction. The submatrix $A_{3,7}$ has 1's in the columns corresponding to each reaction pair (i,j) in each row. $B_3(i) = 1$ for all $i = 1,2,3,\dots,|RE|$.

Row 4 – Normalized flux–gene relationship

We enforce:

$$\frac{v_j - \min_d\{\alpha_{j,d}\}}{\max_d\{\beta_{j,d}\} - \min_d\{\alpha_{j,d}\}} = \hat{g}_j + \varepsilon_j^1 - \varepsilon_j^2 \quad \text{for all } j = 1, 2, 3, \dots, n$$

Rewriting: $$\left(\frac{1}{\max_d\{\beta_{j,d}\} - \min_d\{\alpha_{j,d}\}}\right)v_j - \hat{g}_j - \varepsilon_j^1 + \varepsilon_j^2 = \frac{\min_d\{\alpha_{j,d}\}}{\max_d\{\beta_{j,d}\} - \min_d\{\alpha_{j,d}\}}$$

Where:

- $\hat{g}_j = g_j$ if only one gene is associated with reaction j.
- $\hat{g}_j = q_j$ if the GPR falls under Cases 2–4 (complex rules).

Thus:

- $A_{41}(i,j) = \frac{1}{\max_d\{\beta_{j,d}\} - \min_d\{\alpha_{j,d}\}}$ if $i = j$, else 0.
- $A_{42}(i,j) = -1$ if $\hat{g}_j = g_j$ and it relates to reaction i.
- $A_{43}(i,j) = -1$ if $\hat{g}_j = q_j$ and it relates to reaction i.
- $A_{4,5} = [(-1) \times I_{n\times n} \quad I_{n\times n}]$ (coefficient of ε).

The RHS $B_4(j) = \frac{\min_d\{\alpha_{j,d}\}}{\max_d\{\beta_{j,d}\} - \min_d\{\alpha_{j,d}\}}$ is the scaled minimum flux value for reaction j.

Forming: $A_{4,1}v + A_{4,2}g + A_{4,3}q + A_{4,5}\varepsilon = B_4$.

Row 5 – Gene expression deviation

We have: $g_i - \tilde{g}_i = \delta_i^1 - \delta_i^2$ for all $i = 1,2,3,\dots,k$

or equivalently: $g_i - \delta_i^1 + \delta_i^2 = \tilde{g}_i$

Here:

- $A_{52} = I_{k\times k}$ (coefficient of ggg)
- $A_{54} = [(-1) \times I_{k\times k} \quad I_{k\times k}]$
- $B_5(j) = \tilde{g}_j$, where $\tilde{g}_j$ is the measured relative gene expression (computed via the chosen normalization method: max, mean, or max–min scaling).

Rows 6 and 7 – GPR logic constraints

- Row 6 encodes the logical relationship between g, q, and y based on the transformed GPR rules (Cases 2–4 above).
- Row 7 enforces the summation constraints on the binary $\mathrm{y_i}$ variables to select the minimum or maximum gene expression values in 'AND' and 'OR' logic.

Lower and upper bound of variables in IGM

In the IGM framework, the lower bound (lb) and upper bound (ub) of variables are defined according to their respective types. The reaction flux v_i is constrained by the lower and upper bounds derived from flux balance analysis (FBA). The variables g_i and q_i which represent normalized gene expression levels, take values between 0 and 1. The variables δ_i and ε_i denote the differences between the gene variable value and the measured gene expression data, and between the relative gene variable value and the flux variable, respectively; thus, their ranges are also between 0 and 1. For the binary variables y_i and b_i which can only take the values 0 or 1, are used to determine the minimum and maximum relative gene expression values in the GPR rules and the reaction flow in the reversible model, respectively.

S4 Text. Correlation between gene expression value and value of gene variables across conditions for Data-B

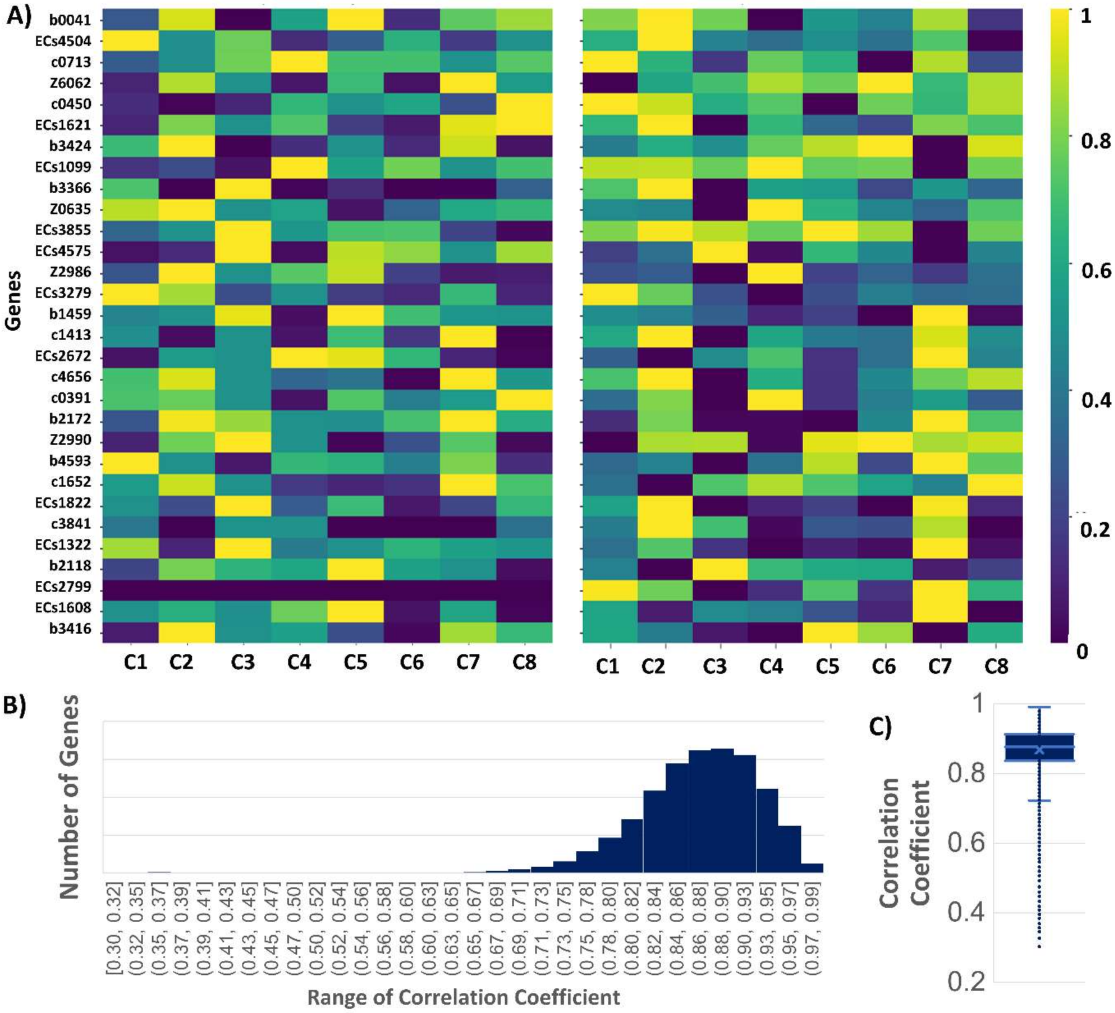

S3 Fig. Relative gene expression values and gene expression variable values of Data-B. (A) Histogram showing the distribution of correlation coefficients between relative gene expression values and gene expression variable values. (B) Box plot of correlation coefficients between relative gene expression values and gene expression variable values.

We randomly selected 30 genes to generate heatmaps comparing the relative gene expression values with the corresponding gene variable values in the IGM framework, thereby illustrating the mapping patterns for Data-B and Data-C (Figures S3A and S4A, respectively). We then calculated the correlation coefficients between the relative gene expression levels and their corresponding gene variable values for each gene. The resulting correlation distributions—shown in Figure S3B for Data-B and Figure S4B for Data-C—are heavily skewed toward high positive values. The box plots in Figure S3C indicate that the majority of genes exhibit correlation coefficients above 0.8, with an average correlation of approximately 0.86 and most values falling within the range of 0.88–0.91. For Data-C, the box plots (Figure S4C) also show consistently high correlations, due to the small number of conditions. The average correlation across all genes for Data-C is approximately 0.91, with most genes falling within the 0.95–0.98 range.

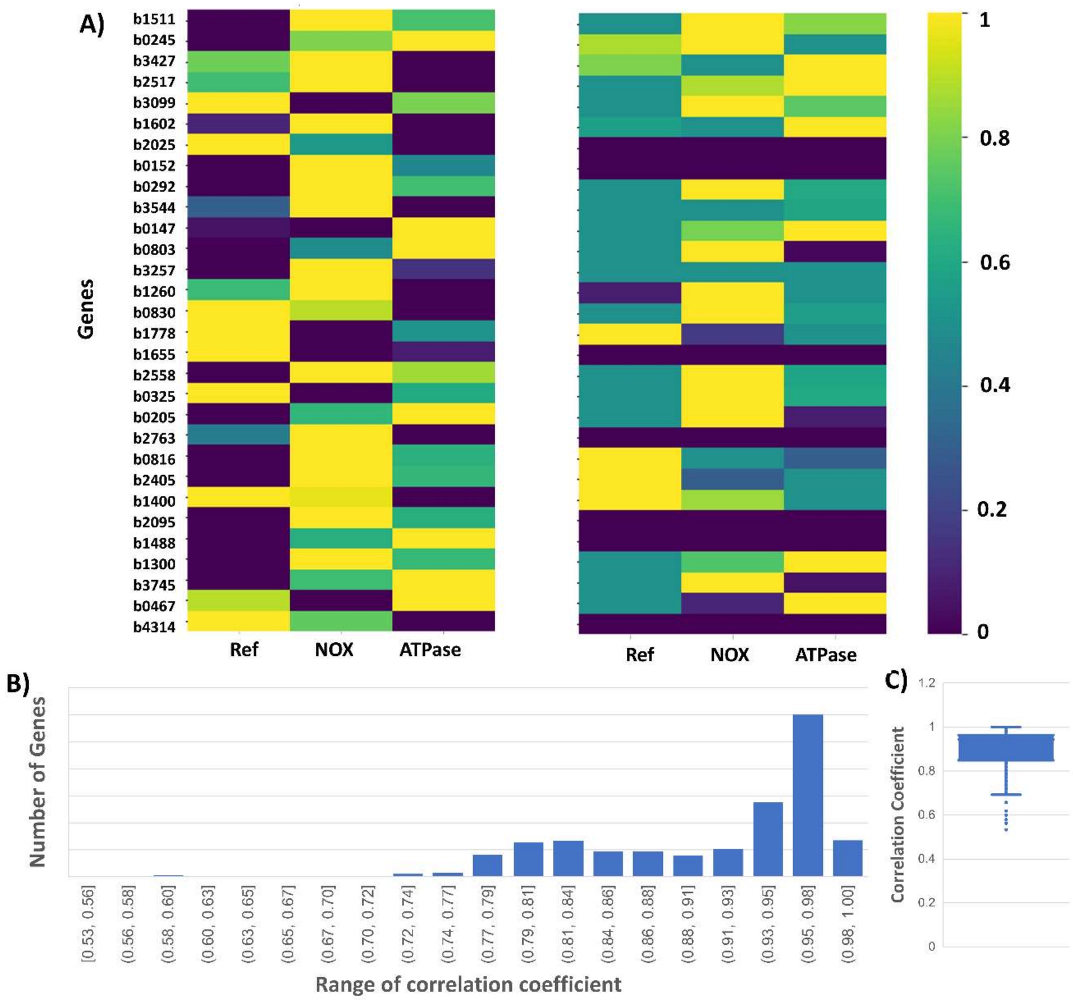

S4 Fig. Relative gene expression values and gene expression variable values of Data-C. (A) Histogram showing the distribution of correlation coefficients between relative gene expression values and gene expression variable values. (B) Box plot of correlation coefficients between relative gene expression values and gene expression variable values.

S5 Text. Relationship Between Flux Values and Gene Variable Values via GPR Mapping

The heatmaps of average flux values in each metabolic subsystem were compared with the corresponding average gene variable values obtained via the GPR mapping. These comparisons for Data-A, Data-B, and Data-C are presented in Figures S5, S6, and S7, respectively. The color patterns in the flux-based and gene-variable-based heatmaps are highly similar, indicating a strong correspondence between reaction fluxes and their associated gene variables across conditions.

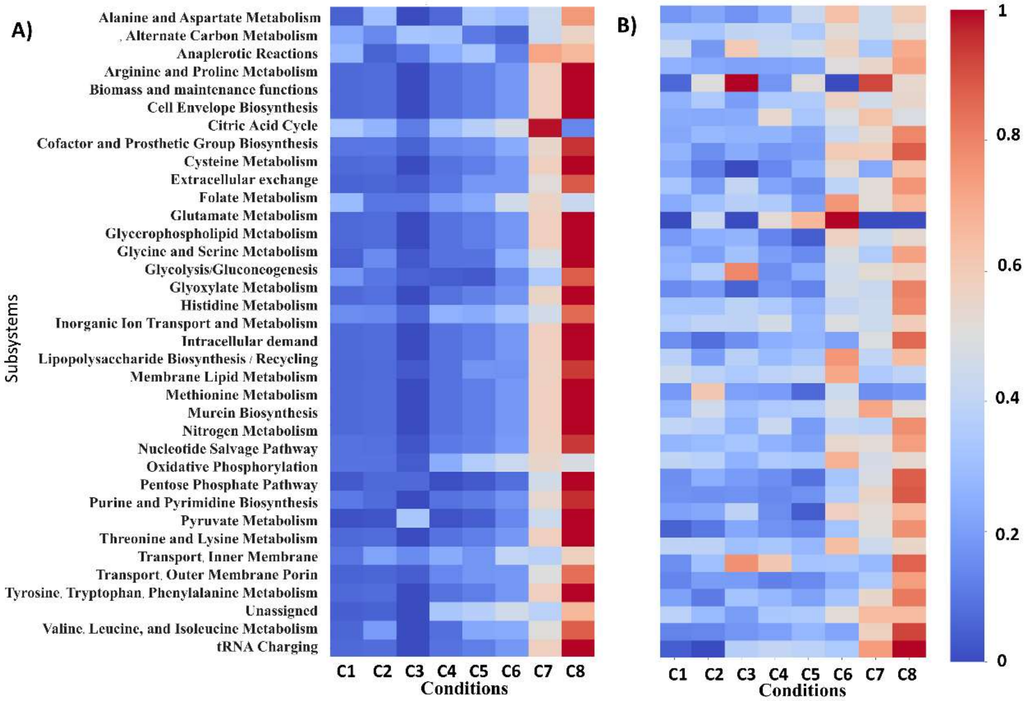

S5 Fig. Relationship Between Flux Values and Gene Variables via GPR Mapping for Data-A. (A) Heatmap showing the row-normalized average flux values in each metabolic subsystem across conditions. (B) Heatmap showing the row-normalized average gene variable values obtained via GPR rules for Data-A (C1 to C8). Conditions C1, C7, and C8 correspond to WT0.2, WT0.5, and WT0.7, respectively. Conditions C2 to C6 represent five single-gene deletion strains: *pgm*, *pgi*, *gapC*, *zwf*, and *rpe*, respectively. Heatmap values range from 0 to 1, with blue indicating values near 0 and red indicating values near 1.

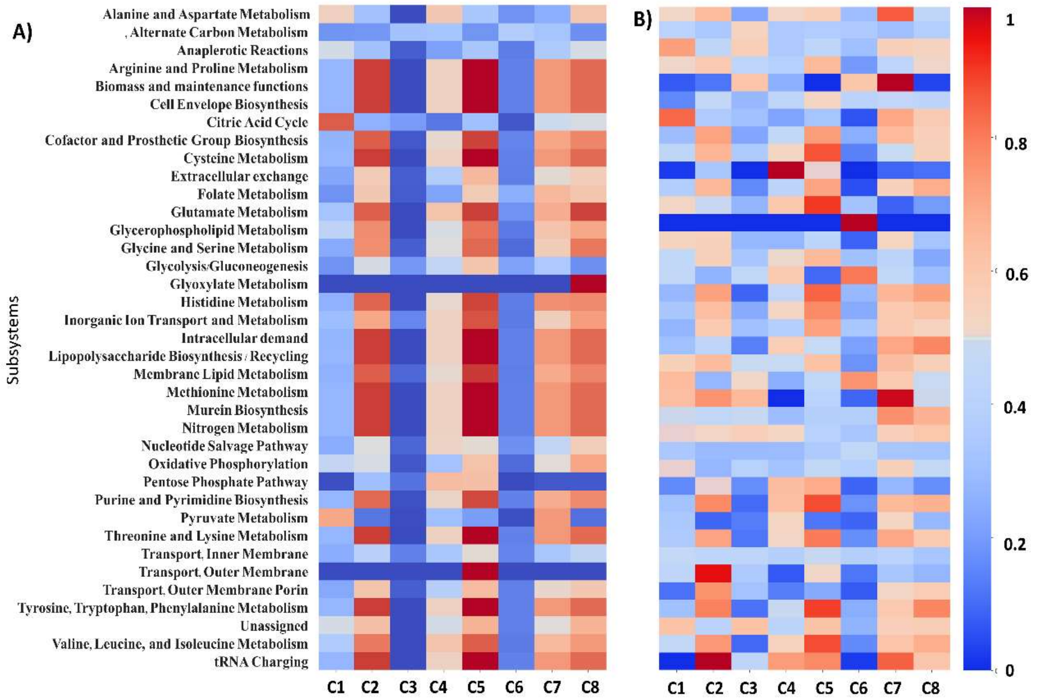

S6 Fig. Relationship Between Flux Values and Gene Variables via GPR Mapping for Data-B. (A) Heatmap showing the row-normalized average flux values in each metabolic subsystem across conditions and (B) heatmap showing the row-normalizing average gene variable value via GPR rule on Data-B: C1 to C8, representing eight carbon sources: acetate, fructose, galactose, gluconate, glucose, glycerol, pyruvate, and succinate, respectively.

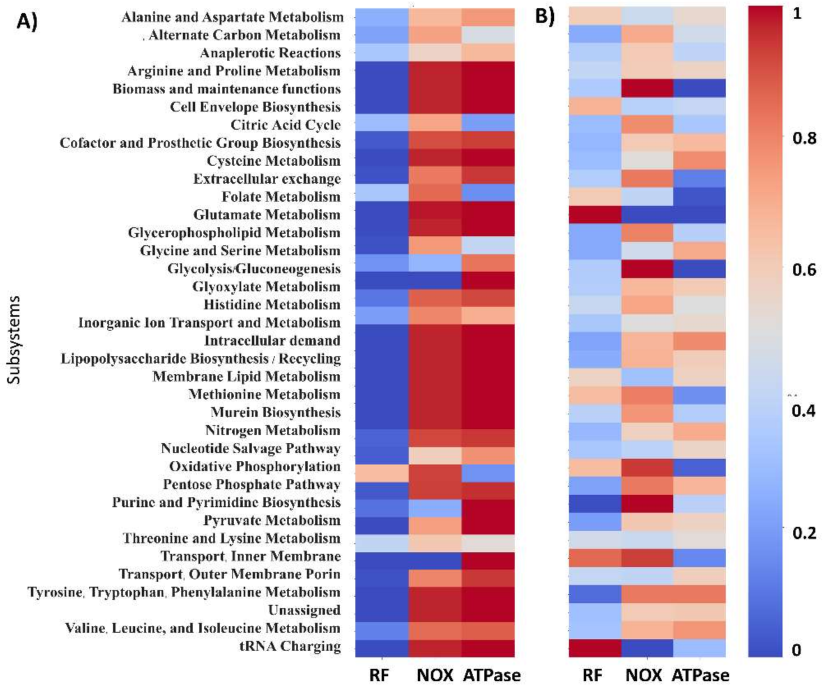

S7 Fig. Flux solution change analysis for Data-C. (A) Heatmap showing the row-normalized average flux values in each metabolic subsystem across conditions. (B) Heatmap showing the row-normalized average gene variable values obtained via GPR rules for Data-C

(C1 to C3). Conditions C1 denotes the reference condition—strains grown in MOPS medium supplemented with glucose during the mid-exponential phase—and two other conditions: an NADH oxidase mutant (C2) and an ATPase mutant (C3). The heatmap values range from 0 to 1, with blue tone indicating values near 0 and red tone indicating values near 1.

S6 Text. Flux Change Analysis

In this study, scatter plots of flux changes for Data-A, Data-B, and Data-C are presented in Figures S9, S10, and S11, respectively. Each figure contains subplots comparing reaction fluxes (in log scale) between every pair of conditions, with the dashed line y=x representing identical flux values in the two conditions. In Figure S8, the fluxes in conditions C1-C7 are generally aligned along the y=x line, indicating that most reactions are similar across these conditions, although a few reactions deviate slightly. In contrast, the fluxes in C7 and C8 (wild-type at 0.5 and 0.7 per hour) deviate substantially from other conditions, with overall higher fluxes. Notably, fluxes in C8 exceed those in C7, likely due to the higher availability of carbon sources in this condition.

In Figure S9, reaction fluxes differ substantially across all conditions, reflecting the use of different carbon sources and their distinct effects on metabolic activity. In Figure S10, most points show that reaction fluxes in C2 (NADH oxidase mutant) and C3 (ATPase mutant) are significantly higher than in C1 (wild-type), highlighting the metabolic adaptations resulting from these gene deletions.

These analyses demonstrate that both genetic perturbations and changes in carbon source significantly influence reaction flux distributions. Importantly, the comparison across wild-type growth rates indicates that higher growth rates are associated with globally increased fluxes, while specific gene knockouts can selectively elevate flux through particular pathways, suggesting compensatory metabolic responses.

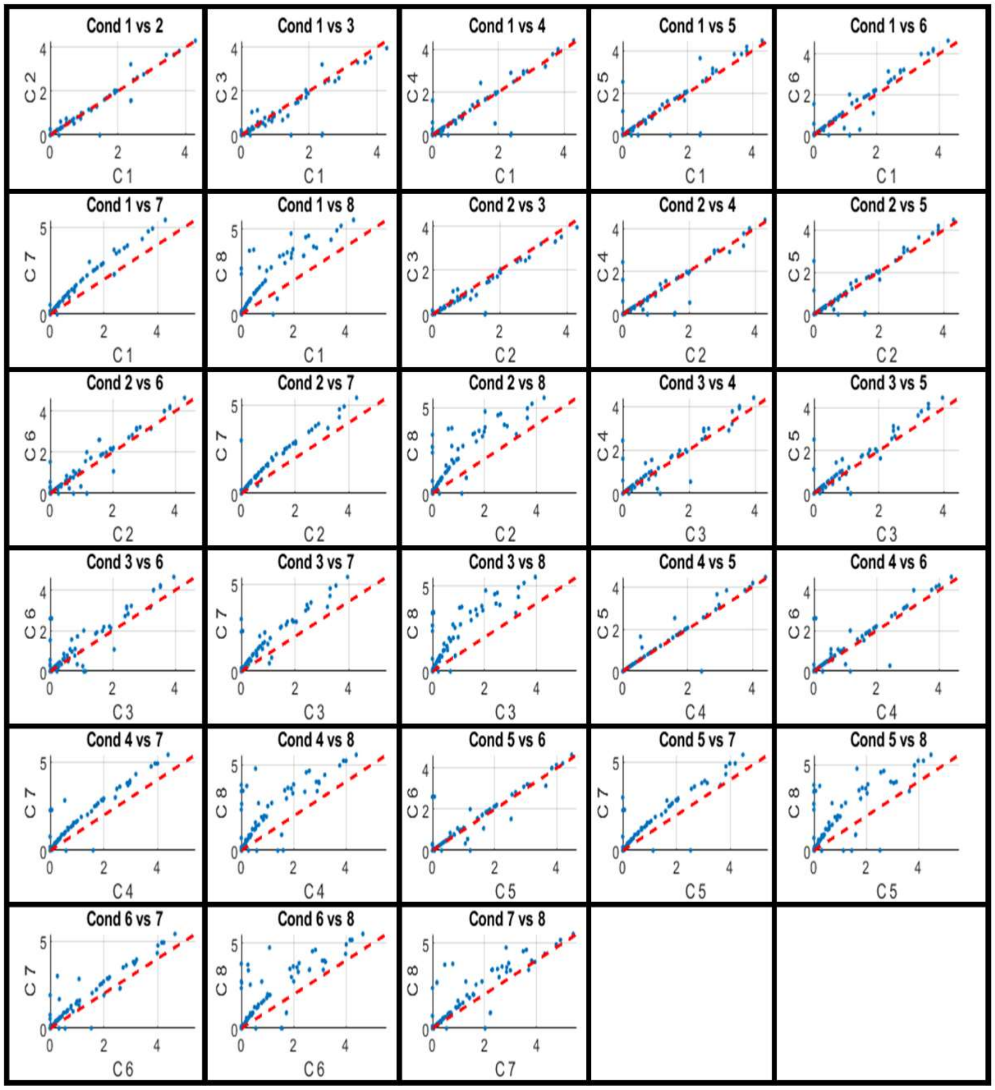

S8 Fig. Scatter subplots of reaction flux changes between pairs of conditions in Data-A (C1–C8). Conditions C1, C7, and C8 correspond to the wild-type strain at growth rates of 0.2, 0.5, and 0.7 per hour, respectively. Conditions C2 to C6 represent five single-gene deletion strains: *pgm*, *pgi*, *gapC*, *zwf*, and *rpe*, respectively. Each subplot compares flux values between a pair of conditions, with the x-axis and y-axis representing the flux values under the two conditions being compared. The red dashed line indicates y=x, highlighting deviations from identical flux values.

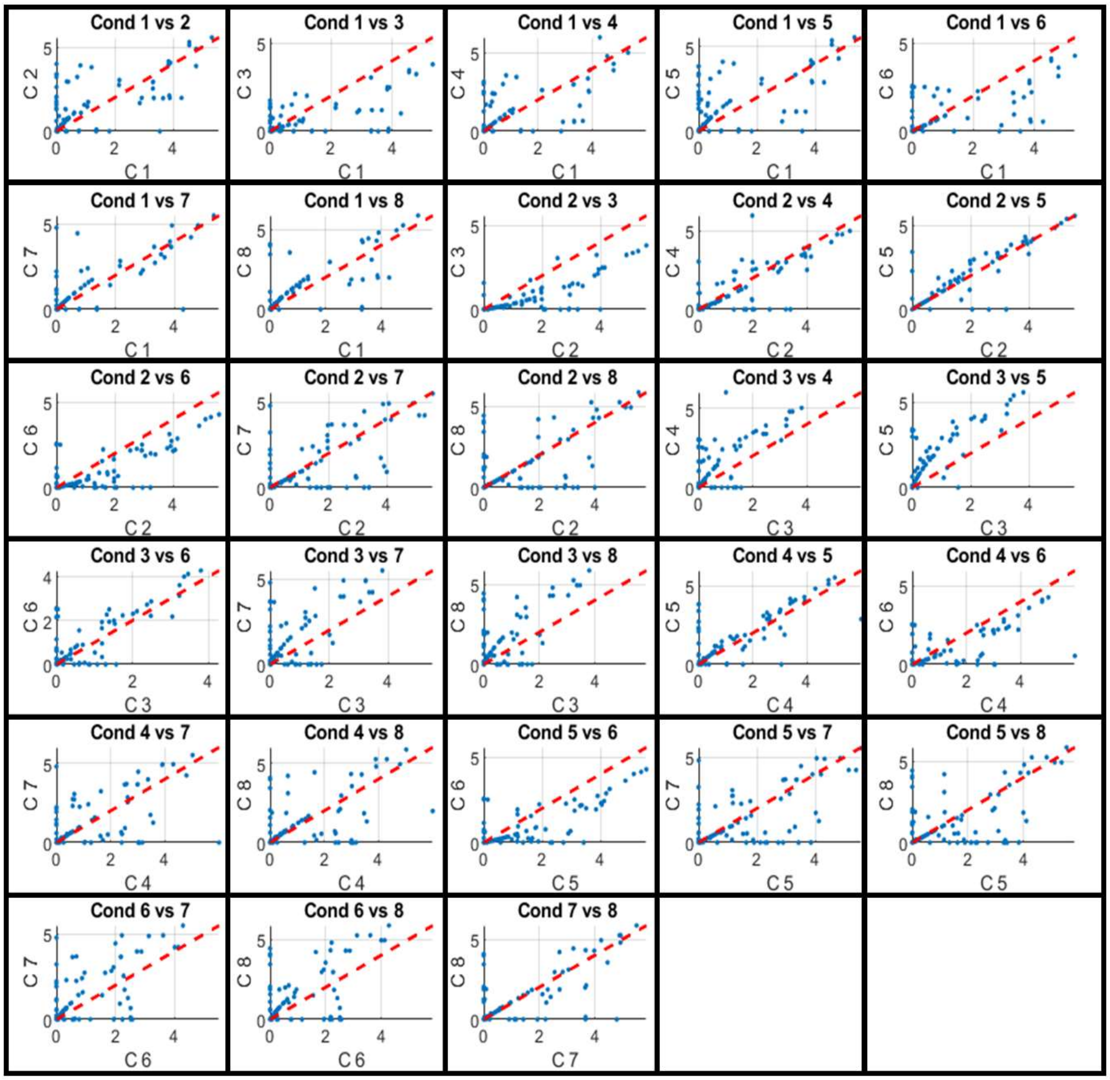

S9 Fig. Scatter subplots of flux changes between pairs of conditions in Data-B: **(C1 to C8)**. Conditions C1-C8, represent eight carbon sources: acetate, fructose, galactose, gluconate, glucose, glycerol, pyruvate, and succinate, respectively. Each subplot compares flux values between a pair of conditions, with the x-axis and y-axis representing the flux values under the two conditions being compared. The red dashed line indicates y=x, highlighting deviations from identical flux values.

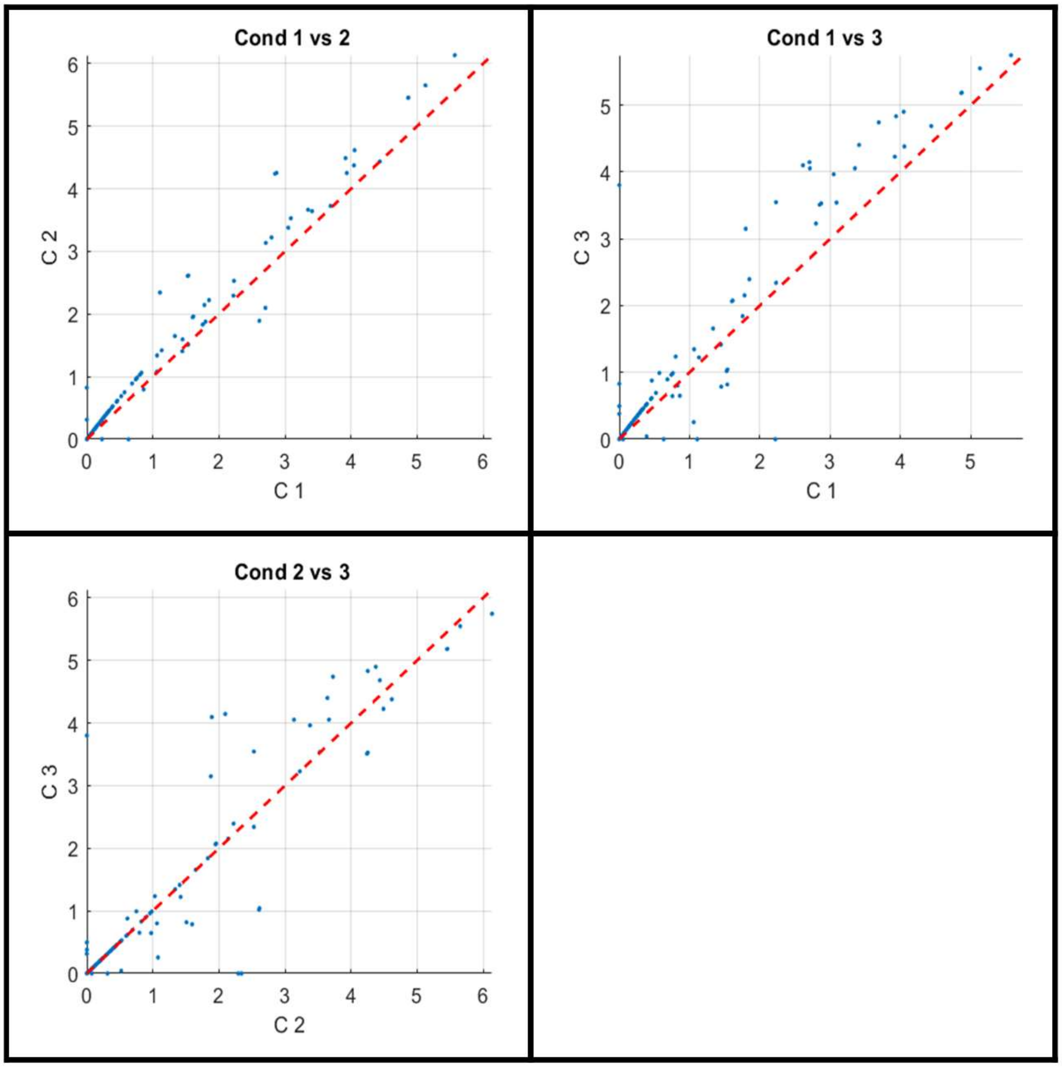

S10 Fig. Scatter subplots of flux changes between pairs of conditions in Data-C: **(C1 to C3)**. Conditions C1 represents the reference condition— strains grown in MOPS medium supplemented with glucose during the mid-exponential phase—and two other conditions: an NADH oxidase mutant (C2) and an ATPase mutant (C3). Each subplot compares flux values between a pair of conditions, with the x-axis and y-axis representing the flux values under the two conditions being compared. The red dashed line indicates y=x, highlighting deviations from identical flux values.

Additional results from the flux change analysis are presented in Figure S11. Panels S8A), S8B), and S8E) show scatter plots (in log scale) of reaction fluxes comparing the glucose carbon source condition (C5) (control) with the glycerol (C6), pyruvate (C7), and succinate (C8) conditions (threat), respectively. Panels S11C), S11D), and S11F) present horizontal bar plots illustrating the top 10 upregulated and downregulated relative flux changes for these same comparisons (C5 vs. C6, C5 vs. C7, and C5 vs. C8, respectively). These visualizations highlight the metabolic reactions most affected by the change in carbon source, both in terms of absolute flux magnitude and relative variation.

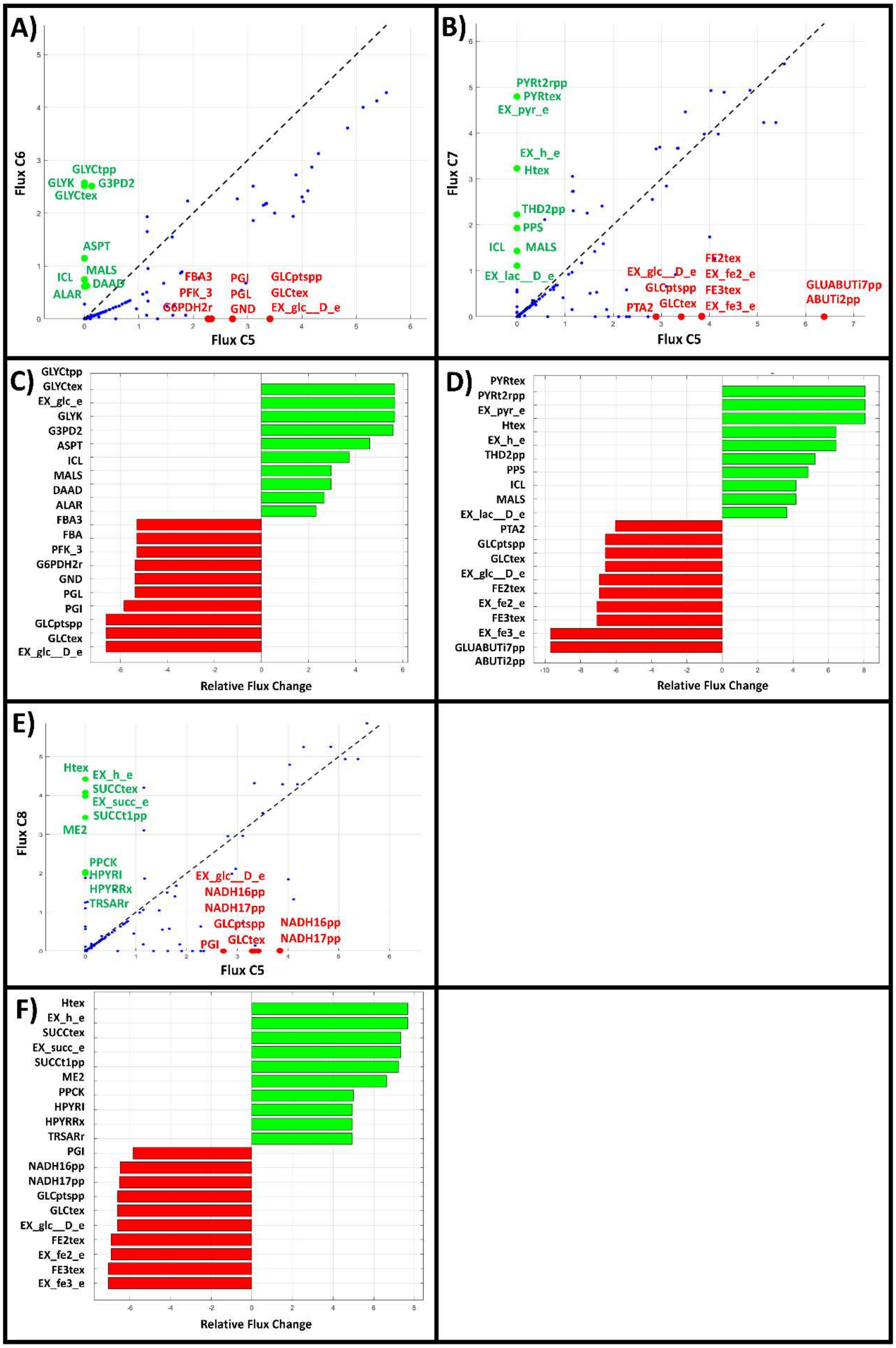

S11 Fig. Flux change analysis in Data-B. (A), (B), and (E) Scatter plots of reaction flux changes comparing the glucose carbon source condition (C5) with three other conditions: glycerol (C6), pyruvate (C7), and succinate (C8). The x-axis represents the flux values (log scale) under the glucose condition, and the y-axis represents the flux values (log-scale) under

the other conditions. The black dashed line indicates line y=x. The green and red nodes highlight the top 10 upregulated and downregulated relative reaction fluxes, respectively, with reaction names labeled; all other reactions are shown as blue nodes. Panels (C), (D), and (F) show horizontal bar plots of the top 10 upregulated (green) and downregulated (red) reactions, along with their relative flux scores.
